# Urge-tic associations in children and adolescents with Tourette syndrome

**DOI:** 10.1038/s41598-022-19685-5

**Published:** 2022-09-26

**Authors:** Jennifer Langelage, Julius Verrel, Julia Friedrich, Alina Siekmann, Ronja Schappert, Annet Bluschke, Veit Roessner, Theresa Paulus, Tobias Bäumer, Christian Frings, Christian Beste, Alexander Münchau

**Affiliations:** 1 Institute of Systems Motor Science, Center of Brain, Behavior and Metabolism, Universität zu Lübeck, Ratzeburger Allee 160, 23538 Lübeck, Germany; 2 Cognitive Neurophysiology, Department of Child and Adolescent Psychiatry, Faculty of Medicine, TU Dresden, Dresden, Germany; 3 Department of Neurology, Universität zu Lübeck, Lübeck, Germany; 4 Cognitive Psychology, Department of Psychology, University of Trier, Trier, Germany

**Keywords:** Neurology, Psychology

## Abstract

Premonitory urges preceding tics are a cardinal feature of Gilles de la Tourette syndrome (GTS), a developmental disorder usually starting during middle childhood. However, the temporal relation between urges and tics has only been investigated in adults. In 25 children and adolescents with GTS (8–18 years), we assess urge-tic associations, including inter-individual differences, correlation to clinical measures, and in comparison to a previously reported sample of adult GTS patients. Group-level analyses confirmed positive associations between urges and tics. However, at the individual level, less than half of participants showed positive associations, a similar proportion did not, and in two participants, the association was reversed. Tic expression and subjective urge levels correlated with corresponding clinical scores and participants with more severe tics during the urge monitor exhibited stronger urge-tic associations. Associations between reported urge levels and instantaneous tic intensity tended to be less pronounced in children and adolescents than in adult GTS patients. The observed heterogeneity of urge-tic associations cast doubt on the notion that tics are directly caused by urges. More severe tics may facilitate anticipation of tics and thereby lead to more pronounced urge-tic associations, consistent with a hypothesis of urges as a byproduct of tics.

## Introduction

Gilles de la Tourette syndrome (GTS) is a neuropsychiatric disorder usually beginning in early to middle childhood^1,2^. According to DSM-5, the diagnosis requires at least two motor and one vocal tic manifesting for a minimum duration of one year with an onset before the age of 18 years^3^. Usually, first symptoms are simple motor tics such as eye blinking or facial tics, starting between the ages of 3 and 8 years^1^. Vocal tics such as repetitive coughing or throat clearing typically develop several years after motor tic onset^2,4–7^. Tic expression varies between patients and fluctuates in severity and appearance intra-individually^2,6,8^. Motor and vocal tics tend to intensify and become more complex over time with a peak of symptom severity usually around 10–12 years^2,5,9,10^. In many cases, symptoms reduce/become less severe during adolescence and can even disappear in adulthood^2,6,11^.

In addition to motor or vocal tics, the majority of adult patients with GTS experience premonitory sensations or urges prior to the actual motor or vocal tic^12–14^. Premonitory urges, which usually develop several years after the first tic symptoms in children, are often described as unpleasant tingling or pressure-like sensations^14,15^. These can present as generalized inner tension or more specifically at body parts involved in an upcoming tic^14–16^. Many patients with GTS report that premonitory sensations are reduced by tic execution, resulting in a feeling of relief^17,18^. Based on this, it has been proposed that the execution of tics may be reinforced by alleviating the unpleasant premonitory sensation, thus creating a negative reinforcement cycle with urge as a primary driving factor^19–22^. This view is supported by experimental studies demonstrating higher overall urge intensities during periods of instructed tic suppression^23^ as well as an alleviation of urge intensity during periods without the need to suppress tics^19^. For a more fine-grained temporal analysis of urge-tic associations, our group developed the continuous-time urge-monitor^12^, in which participants are instructed to continuously indicate their currently experienced premonitory urge. Motor and vocal tics are captured by synchronized video recordings for subsequent (offline) second-by-second tic ratings. Assessing the temporal relationship between continuous urge ratings and concurrent tics in adult patients with GTS, acquired using the urge monitor, confirmed an increase–decrease pattern of urges relative to the time of tic execution as well as a positive association between instantaneous urge intensity and tic occurrence^12^.

However, evidence from both experimental and epidemiological-developmental studies casts doubt on a straightforward causal relation with tics being primarily driven by urges. Positive urge-tic associations reported by Brandt et al.^12^ are based on group-level analyses, for which urge and tic times series from all participants were concatenated to increase the statistical power for detecting associations and assess the average urge-tic pattern across participants. However, if tics were primarily driven by urges, pronounced positive urge-tic associations should also be detectable when restricting these analyses to data from individual participants. This was done in a recent study using the same assessment tool, the urge monitor, revealing substantial inter-individual heterogeneity in urge-tic associations in an adult sample of patients with GTS^24^. While this study confirmed a positive association between subjectively reported urge and video-based tic ratings at the group level, i.e., higher likelihood of tics and higher tic intensity during time intervals of elevated urge, analyses at the individual level revealed that about a third of participants did not show such an association or even showed reduced tic expression during times of elevated urge, i.e., a negative urge-tic association. Second, if tics were primarily driven by urges, urges and tics should appear simultaneously during development, or urges should even appear before tics develop. However, as mentioned above, premonitory sensations usually occur several years after tic onset^4,7,15^. This has led to the proposal that urges might develop as a consequence of increasing (bodily) self-awareness, including awareness of tics, and therefore are more likely an adaptation to experiencing tics rather than directly causing tic execution^15,18,24^.

Given the developmental nature of GTS and divergent views on the relation between urges and tics, the current study investigates the presence, strength, and heterogeneity of urge-tic associations in children and adolescents with GTS. The aims of the study are threefold. First, we provide the first temporally fine-grained analysis of the relation between premonitory urges and tics in a sample of children and adolescents using an established tool, the urge monitor, which combines continuous urge ratings with synchronized video recordings of tics^12,24^. Second, inter-individual differences in tics, urge ratings and urge-tic associations are scrutinized by assessing their inter-relation and correlation with clinical data. Third, the current developmental sample was compared to previously acquired data from an adult sample using the same paradigm and analytic methodology^24^. If urges were the driving force for tics, urge-tic association can be expected to be “purer”, i.e., more consistent and more pronounced, in children and adolescents compared to adults, where voluntary tic suppression has been found to lead to a de-coupling of urges and tics^12^. Contrary to this view, we hypothesize that urges may be a “byproduct” of a developing ability to anticipate tics, leading to the prediction that urge-tic associations are more pronounced in adults compared to children and adolescents.

## Methods and materials

### Participants

We examined 25 children and adolescents (8 females and 17 males) aged from 8 to 18 years (mean ± SD: 13.7 ± 2.7 years). The inclusion criteria were: being 18 years old or younger and having a clinical diagnosis of GTS based on DSM-5 criteria^3^ Exclusion criteria were: relevant psychiatric co-existing conditions apart from obsessive–compulsive disorder (OCD) or attention deficit hyperactivity disorder (ADHD), co-existing neurological disorders such as epilepsy, a change of medication within the last four weeks prior to testing, or an intelligence quotient (IQ) below 80 (in our sample: 105.8 ± 9.9, 88–123). Patients with co-existing OCD and ADHD were not excluded because these are common in GTS^1,25,26^ and we wanted to investigate a representative sample. IQ was assessed using the German version of the short form of the Wechsler Intelligence Scale for Children (HAWIK-IV) for patients aged from 8 to 15 years^27^. Older patients were tested using the short form of the Wechsler Adult Intelligence Scale^28,29^.

Experimental data from additional 11 children and adolescents were acquired based on the inclusion and exclusion criteria but could not be used in the analysis for the following reasons: poor video quality (N = 8), technical problems with urge data acquisition (N = 2), incorrect task execution (N = 1). Patients were recruited from the GTS specialty clinic in the Department of Pediatrics at the University Medical Center Schleswig–Holstein, Campus Lübeck and the outpatient clinic of the Department of Child and Adolescent Psychiatry at the University Medical Center Dresden.

According to the psychiatric assessment (MINI/MINI-KID^30,31^), eight patients had co-existing OCD and two had co-existing ADHD. In addition, depressive episodes in the past (N = 2), conduct (N = 1) and anxiety disorder (N = 1), agoraphobia (N = 1), manic episodes (N = 1), and psychotic disorder in the past (N = 1), were identified. None of these findings were clinically relevant at the time of investigation. One patient had polyneuropathy. Four participants took centrally acting medication (three were taking tiapride, of whom one took atomoxetine additionally and one participant took methylphenidate). One patient had used cannabis in the past. Details on the clinical assessment and clinical data are provided in Sect. 1 of the Supplementary Materials.

The data from the children and adolescents of the current study are compared to data on urge-tic associations in 21 adult patients with GTS (10 female, 11 male; 30.5 ± 10.6 years, 18–50 years) previously published^24^, the data, analysis scripts and experimental software of which are publicly available (https://osf.io/k9z5c/). Clinical assessment and the data analysis pipeline are aligned between the studies. The rating scheme for the adult sample included additional information on body region and tic onset and offset. However, the subset of the rating schemes used for the urge-tic analysis is identical between the two studies. In particular, the same rating scale was used (see Sect. 2.2 of Supplementary Material) and the raters for the present study were trained and supervised by the raters of the previously acquired adult data^24^ until high inter-rater reliability was achieved between all four raters.

Clinical inclusion and exclusion criteria were determined before data acquisition. Exclusion criteria concerning data quality were determined during preliminary data inspection but prior to statistical analysis. The Ethics Committee of the TU Dresden (EK 359092017) approved this study and written informed consent of parents/legal representatives as well as minor participants was obtained prior to participation. The study was conducted in accordance with the declaration of Helsinki.

### Real-time urge and tic assessment

**Figure 1:**
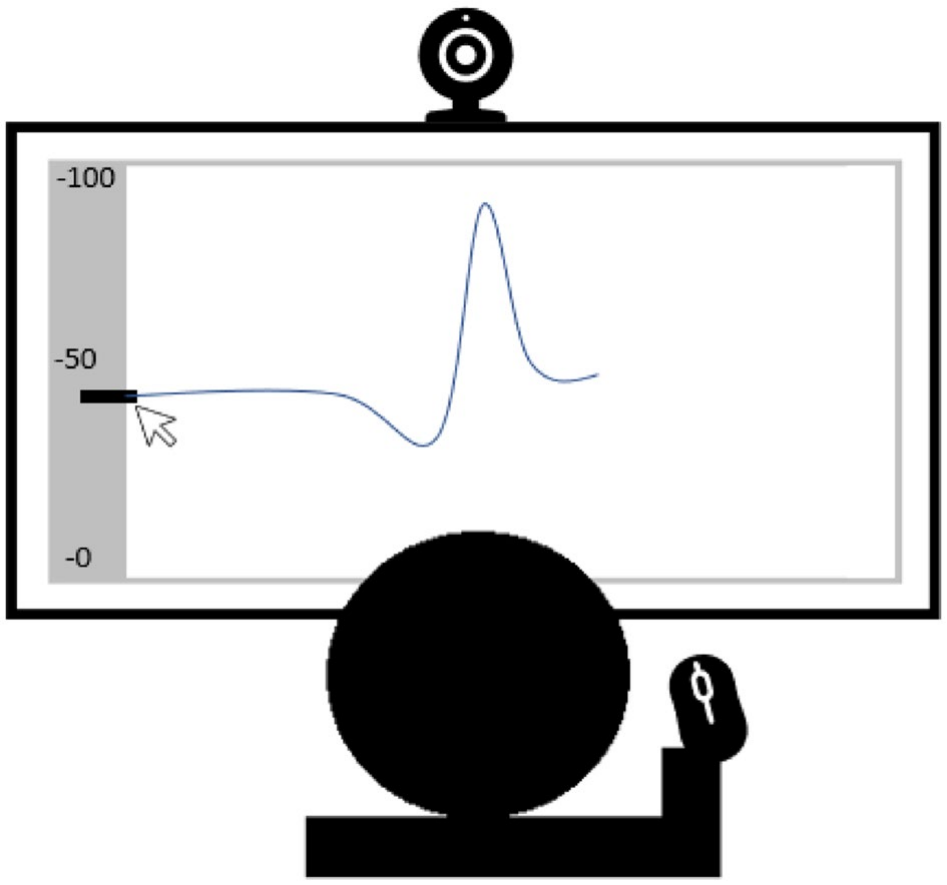
While performing the urge monitor task, participants were seated in front of a computer screen, using a computer mouse to continuously report their current subjective urge level on a vertical scale (0–100). Participants’ upper-body movements were video-recorded for subsequent video-based tic ratings.

The urge monitor was implemented in Python 2.7, using the PsychoPy toolbox version 1.83.04^32^. Participants were comfortably seated in front of a computer screen (Lübeck; Iiyama B2780HSU-B1, 1920 × 1080 pixels; Dresden: ASUS VG248QE, 1920 × 1080 image resolution) and instructed to continuously indicate their currently experienced urge intensity by controlling the vertical position of a cursor on the screen using the computer mouse. In addition to the slider indicating the current urge, the recent urge history (3 s) was shown as a continuous curve (see Fig. 1). The urge monitor display, spanning about 6° visual angle horizontally and vertically, was updated at a frequency of 30 Hz. Urge data were recorded at 10 Hz for subsequent analysis. Participants received standardized instructions, indicating possible manifestations of premonitory sensations or urges (e.g., "similar to the feeling preceding sneezing"; "general tension"; "anything that might signal a tic") and defining the scale between 0 ("no feeling of urge") and 100 ("strongest urge possible/imaginable").

Participants were instructed not to suppress their tics. Subsequently, participants were briefly familiarized with the task (30 s practice run) and then performed the actual task (300 s). The initial slider position was set to 50% of the intensity scale; data recording (for 300 s) started 5 s after task onset to give participants time to indicate their current urge level.

Motor tics involving the head, face, torso, arms, hands and vocal expressions of the children/adolescents were recorded during the five minutes urge monitor task. In Lübeck, a webcam was used (Logitec c930e, 1920 × 1080 pixels at 30 Hz) recording videos frontally. In Dresden a camcorder (Panasonic HC-V757-K Camcorder, 1920 × 1080 pixels at 30 Hz) was used. Beginning and end of the urge monitor task were marked by audio signals used to synchronize urge data with the video recordings (see Supplementary Material, Sect. 2.1, for further details, including technical problems with audio recordings).

### Video-based tic ratings

Video-based tic ratings were based on the same criteria as in the previously published study with adult GTS patients^24^. Motor and vocal tics were rated from video recordings using the freely available video coding software Datavyu (stable Version 1.3.7)^33^. The relevant (urge-synchronized) part of the video recording was divided into 300 1s-intervals for the tic rating in Datavyu. For each interval, two independent raters assessed the presence/absence of motor and vocal tics and defined the overall intensity of tics occurring in this segment. Intensity of motor tics were defined on a scale from 1 (very mild, could be a normal movement, e.g., a short sniff) to 8 (very severe, involving risk of self-injury, e.g., hitting one’s face). The scale for vocal tics ranged from 1 (very mild, could be a spontaneous physiological sound) to 7 (severe, e.g., multiple coprolalic words, noisy sound) (Supplementary Table S3, Supplementary Material). Because of more frequent occurrence of spontaneous non-tic movements in children in comparison to adult patients, the two raters pre-viewed the video recordings to agree on non-tic movements present in individual participants (e.g., chair motion, self-touch) prior to the actual tic rating. Furthermore, all videos and data from the urge monitor task were checked for any indications of not executing the task properly, leading to exclusion of one patient. Note that vocal tics could not be rated in participants with missing audio recording (N = 8; after exclusion of one participant not executing the task properly).

**Figure 2:**
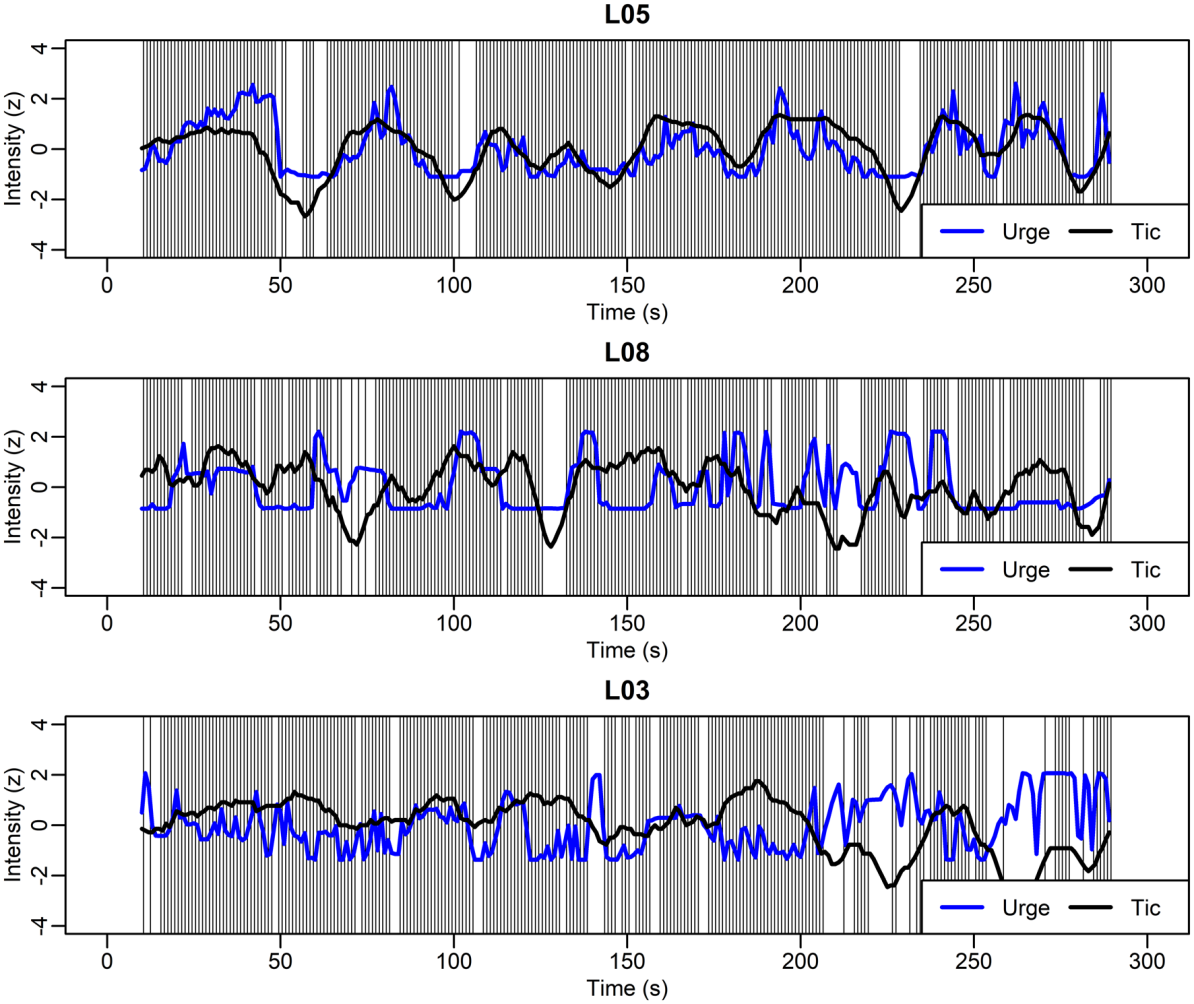
Individual urge-tic time series from three different patients, one with a clearly positive urge-tic association (L05, upper panel), one with no systematic association (L08, middle panel), and one with a negative urge-tic association (L03, lower panel). The plots show subjective urge (blue line), instantaneous tic intensity (black line, central moving average of length 11 s) and times of tics (vertical light-gray lines).

As in the previous study with adults^24^, motor and vocal tic intensities were combined to a single intensity score for each 1s-interval by taking the maximum of the two scores. Inter-rater reliability was assessed using Pearson’s correlation for presence/absence of a tic as well as tic intensity. As an additional quality check, the percentage of 1s-intervals, in which any tic was rated was correlated with the tic frequency determined in the RUSH video protocol, a clinical tool comprising video-based assessment of tic intensity and frequency under standardized conditions (see Supplementary Material, Sect. 1.1). For further analysis, tic presence/absence data from the two raters were combined by logical conjunction (tic present if scored by at least one rater) and tic intensities were averaged for each 1s-interval. Moreover, a continuous time series representing the “instantaneous tic intensity” was computed as a central moving average (window size 11 s) of rated tic intensities per 1s-interval (Fig. 2).

### Statistical analysis

All data processing and statistical analyses were performed in R (version 3.6.2)^34^. The threshold for null-hypothesis significance testing was p < 0.05. Correlation analyses were corrected for multiple comparisons as reported below.

The first and last 10 s of urge and tic time series were not included in the analyses to avoid boundary effects and to give the participants additional time to adjust their current urge level on the scale. Subsequently, the following descriptive statistics of urge and tic occurrence were calculated for each subject: The mean and standard deviation of subjective urge intensities (hereafter referred to as mean and SD urge, respectively), tic frequency (percentage of 1s-intervals with any tic) and the average tic intensity (exclusively for 1s-intervals with any tic).

#### Urge-tic association

Individual time series (urge, tic intensity) were z-standardized (to mean 0, variance 1) per participant prior to the following analyses. The association between urge intensity and tics was analyzed using three statistical approaches: logistic regression (predicting tic from urge), linear regression (urge intensity at tics), and correlation (between urge intensity and instantaneous tic intensity). These analyses were performed at the group level to describe the overall relationship across participants using random effects models (glmer and lmer from R package lme4)^35^ with maximal random effects structure^36^. For urge and tic intensity as predicted variables, z-standardization resulted in non-convergent or singular model fits, presumably due to the elimination of between-subject intercept variance. As in the previous study in adults^24^, this problem was resolved by restricting the analysis to a smaller time window (50–250 s).

Corresponding analyses were performed separately for each participant, using logistic or linear regression (glm, lm in R) and correlation analyses at the individual level (now using the entire urge/tic recording, with only the first and last 10 s removed). Coefficients from the logistic and linear regression as well as the correlation analysis were computed to quantify individual urge-tic relationships, to assess correlation with clinical measures, and for comparison with an adult sample^24^.

#### Inter-individual differences

Pearson´s correlations were calculated between clinical scores and measures originating from the urge monitor task, i.e., measures related to tics (tic frequency, mean tic intensity), measures related to the urge (mean and SD of urge time series) and measures related to the urge-tic association (logistic and linear regression and correlation coefficients). In addition, we assessed correlations among the measures of the urge monitor. For each clinical score, statistical analyses were adjusted for the number of urge monitor measures of each type (tic: 2, urge: 2, urge-tic: 3) using Bonferroni-Holm correction^37^.

#### Comparison between age groups

To gain insight into developmental changes, data from the current sample of children and adolescents were compared to previously published data from an adult sample^24^. This was done by applying two-sample Welch t-tests (allowing for unequal variance) to the individual measures of urge, tic and urge-tic associations described above. Based on the hypothesis that urges develop as an adaptation to tics, leading to the prediction of more pronounced urge-tic associations in adults compared to children and adolescents, the strength of urge-tic associations was compared using one-sided hypothesis tests.

## Results

Exemplary time series data from three participants are shown in Fig. 2. As discussed in more detail below, these represent the observed patterns of a positive urge-tic association, no significant association, and even a negative association.

### Descriptive summary of urge and tic data

A high inter-rater reliability for presence/absence of a tic (median r = 0.71, range 0.5–0.87) and for tic intensity (0.80, range 0.63–0.94) was found. Correlation analysis between tic counts per minute in the RUSH Protocol and tic frequency (%) from the urge monitor revealed a positive correlation (r = 0.62, p = 0.001) despite substantial differences between the protocols of both measures.

Subjective urge monitor ratings revealed substantial variability between individuals. Across participants, the mean urge (rated on a scale from 0 to 100) was 13.0 (range 0.03–58.24) with a mean variation (SD) of 14.89 (0.27–45.81). In the video recordings, tics were noted on average in 61.4% of 1 s-segments (10.67–98.33%) and rated with a mean tic intensity of 2.26 (1.31–3.39). Detailed individual urge and tic data from the urge monitor are provided in the Supplementary Material (Supplementary Table S4 and individual urge-tic time series in Sect. 7).

### Urge-tic association in children and adolescents with GTS

A significant positive association between urge intensity and tic occurrence/intensity was found in each of the three group-level analyses (across all patients). Logistic regression analysis, predicting tic occurrence from z-standardized urge intensity (also included as random slope), revealed a positive effect (β = 0.44, OR = exp(β) = 1.55) of urge intensity on tic occurrence (χ^2^(1) = 7.029, p = 0.008). Linear regression, predicting (z-standardized) urge from tic occurrence (also included in the random effect structure), indicated that urge was elevated (by about 0.20 SD) in tic intervals (β = 0.20, χ^2^(1) = 7.39, p = 0.007). Finally, a random effects linear regression (with urge intensity as random slope) showed a positive effect of urge intensity on instantaneous tic intensity (β = 0.17, χ^2^(1) = 6.64, p = 0.01).

Nine participants indicated not experiencing premonitory urges in the clinical assessment. We decided not to exclude these participants from the study as the instructions of the urge monitor appeared to make sense to each of them. Moreover, we considered it possible that the urge monitor may provide additional, possibly more fine-grained information about participants’ premonitory sensations than a clinical questionnaire (PUTS). Restricting the above group-level analyses to these nine participants revealed significant urge-tic associations for the random effects logistic regression (β = 0.32, OR = exp(β) = 1.38, χ^2^(1) = 8.11, p = 0.004) and linear regression (β = 0.21, χ^2^(1) = 18.29, p < 0.001; the random slope had to be removed to avoid a singular model) fit but not for the correlation between urge and tic intensity (p > 0.2).

The inter-individual heterogeneity of urge-tic associations is illustrated with data from three representative participants in Fig. 2. Patient L05 (top panel) exhibited a pronounced positive urge-tic association: Both the frequency of tic occurrence (density of vertical lines, each indicating tic occurrence in a given 1 s-interval) and the instantaneous tic intensity (black curve) increase and decrease with continuously reported subjective urge intensity (blue curve). In contrast, no such association is evident in the middle panel (L08) and the association even appears to be reversed in the bottom panel (L03), with reduced tic frequency and intensity during periods of elevated urge.

**Figure 3:**
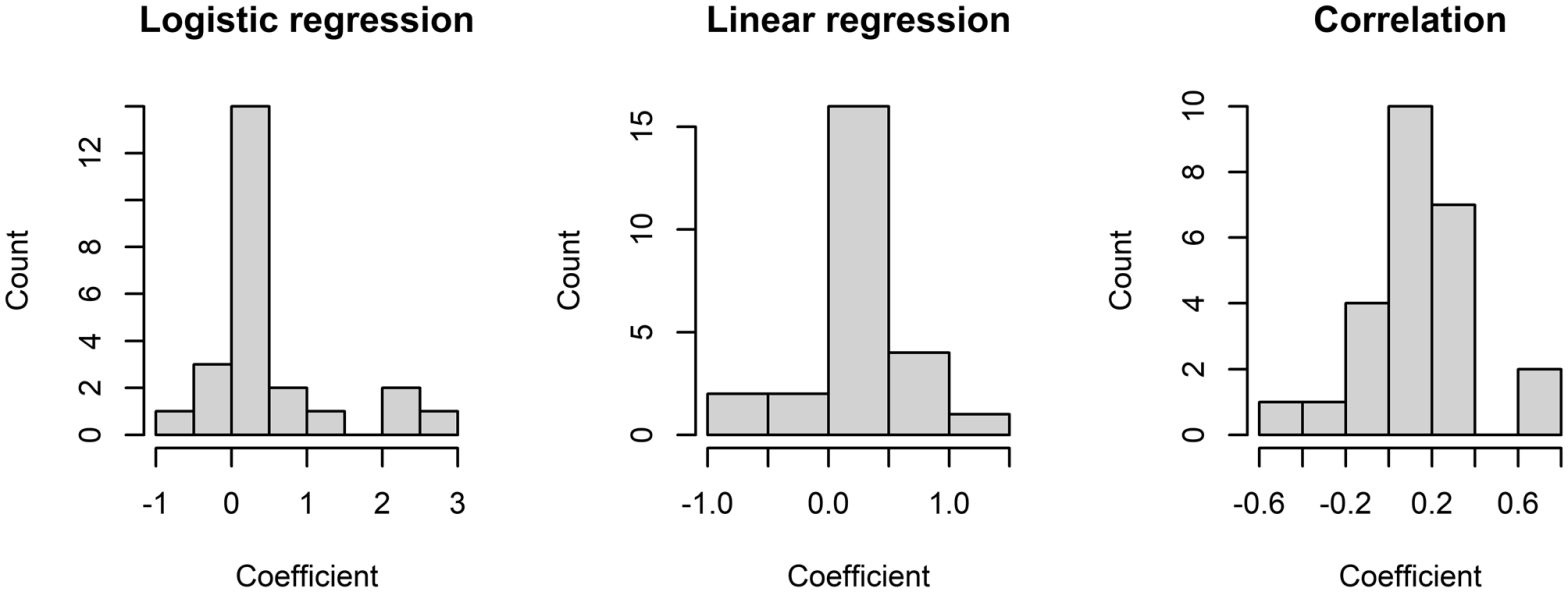
Distribution of measures (regression/correlation coefficients) of urge-tic association in children and adolescents. More positive values indicate a stronger positive urge-tic association, with more/more intense tics at times of elevated urge. Negative values indicate a reversed urge-tic pattern. Values near 0 indicate no or only a weak association.

Individual data and time series plots for all participants can be found in the Supplementary Materials (Supplementary Table S5 and Sect. 7). The distributions of measures of urge-tic association (logistic and linear regression coefficient; correlation coefficient) are shown in Fig. 3. According to the per-participant analysis, 40–48% (depending on the measure) of participants showed a significant positive association between urges and tics, in line with the group-level analyses reported above. For the logistic regression analysis, predicting tic occurrence from urge, the measures of urge-tic association ranged between β =  −0.68 (OR 0.51) and β = 2.57 (OR 13.1), with 11 participants showing a significant positive association, 12 no significant association and 2 a significant negative association between urge intensity and tic occurrence. For the linear regression, comparing (z-transformed) urges at times of tics vs. times in absence of tics, the regression coefficient $\beta$ ranged between −0.67 and 1.29 (positive: 10, n.s.: 13, negative: 2). For the correlation between urge and instantaneous tic intensity, correlation coefficients ranged between -0.45 and 0.68 (positive: 12, n.s.: 11, negative: 2).

### Correlation analyses

Complete results from the correlation analyses are provided in the Supplementary Material (Sect. 5, Figures and Tables S6, S7, S8). Tic measures obtained from the urge monitor video ratings showed positive associations with RUSH total score, RUSH-based tic frequency, and YGTSS (r between 0.54 and 0.72). Urge intensity and variation (mean and SD) from the urge monitor showed positive correlations with RUSH total, RUSH tic frequency, YGTSS, and PUTS, as well as tic frequency (%) and mean tic intensity from the urge monitor (r between 0.48 and 0.66). Positive, though weaker associations of urge measures were also found with GTS-QoL, Conners (IA) and Conners (HI). All three measures of urge-tic association showed correlations with mean tic intensity from the urge monitor (r between 0.46 and 0.62). The fact that neither urge intensity and variation nor tic frequency (%) correlated with measures of urge-tic association is in line with corresponding control analyses in the adult sample^24^ and indicates that the urge-tic associations reported here are not confounded with inter-individual differences in the amount of tics or use/interpretation of the urge scale.

### Comparison to adults with GTS

**Figure 4:**
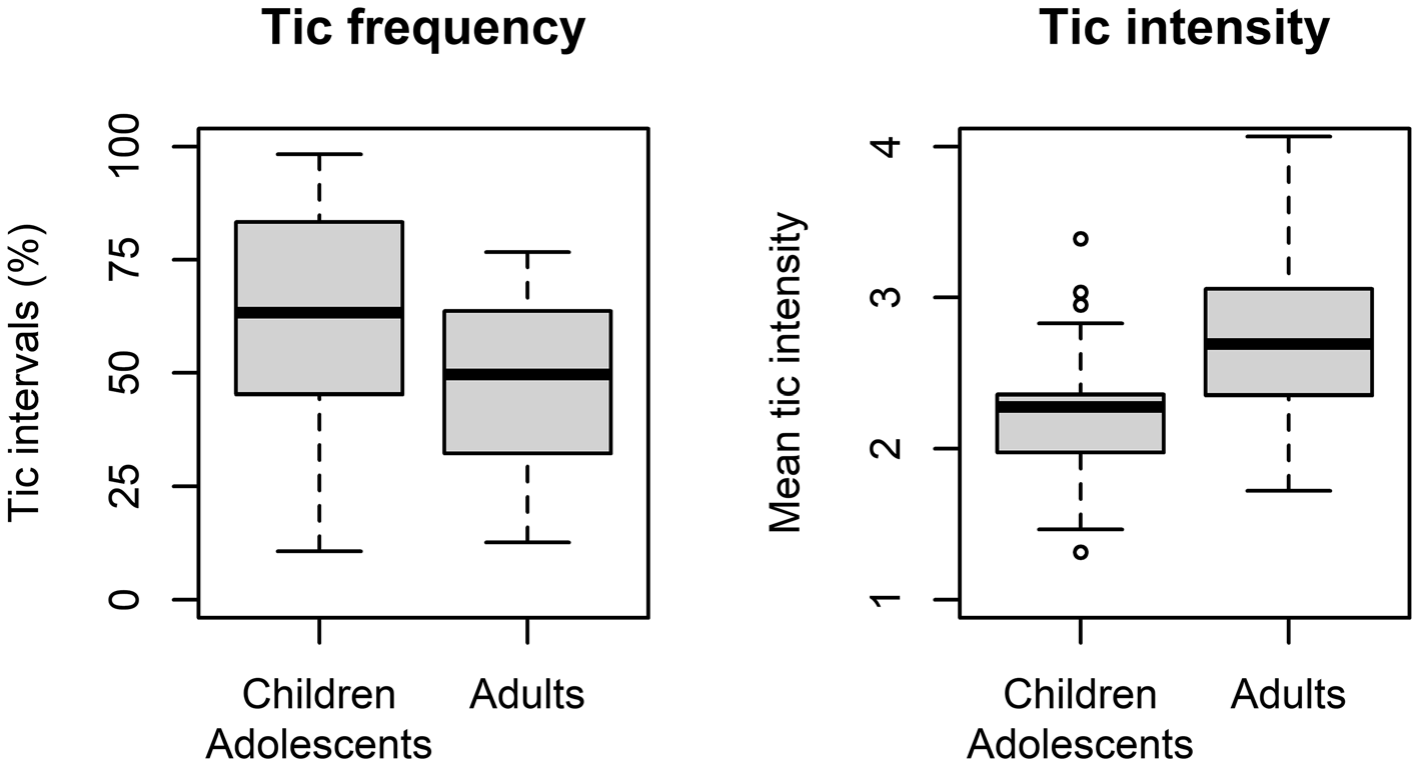
Comparison between age groups for tic-related measures from the urge monitor. Boxes indicate inter-quartile range and median, whiskers extend to values up to 1.5 IQR beyond first and third quartile, respectively. Values outside this range are shown as individual points.

**Figure 5:**
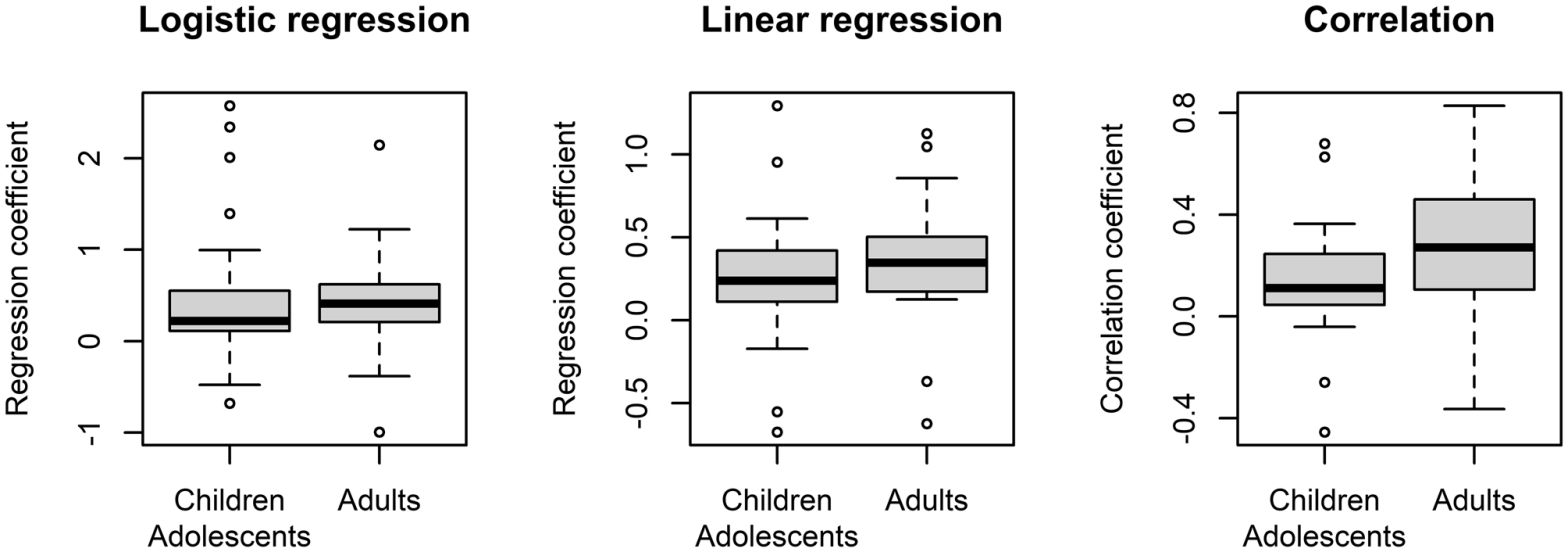
Comparison between age groups for measures of urge-tic association from the urge monitor. Boxes indicate inter-quartile range and median, whiskers extend to values up to 1.5 IQR beyond first and third quartile, respectively. Values outside this range are shown as individual points.

Compared to previously reported data from adult sample^24^, children and adolescents showed a higher tic frequency (%) (t(43.6) = 2.18, p = 0.035) but, on average, less intense tics (t(39.1) = − 2.95, p = 0.005) (see Fig. 4 and Supplementary Material, Table S9). Urge intensity and variation (mean, SD) did not show a systematic difference between age groups (p > 0.28). For measures of urge-tic association, individual logistic and linear regression coefficients did not differ between age groups (two-sided test: p > 0.4, one-sided tests: p > 0.2) while a marginally significant difference was found for correlation coefficients (t(38.23) = − 1.69, two-sided test: p = 0.10, one-sided test “adults > children”: p = 0.05), indicating weaker urge-tic associations in children and adolescents compared to adults (Fig. 5). While consistent with our prediction (see end of “Introduction”), this finding needs to be interpreted with caution, as the group difference was present only in one out of three measures of urge-tic association.

As noted above, vocal tics could not be rated in eight participants of the present sample due to missing audio recording. According to the RUSH protocol ratings, on average 4.3% (SD 2.8%) of tics were vocal in the eight participants with missing audio recording (across all participants: 6.0%, SD 6.6%). Still, this could systematically affect urge-tic associations in the present sample and bias comparisons with the adult sample. To control for this, all group comparisons were repeated including only the non-vocal tics. Restricting the analyses to non-vocal tics only had a minimal effect on the group comparisons, confirming no group differences for the logistic and linear regression analysis (p > 0.4) and a marginally group difference for the correlation between urge and tic intensity (t(38.25) = − 1.68, two-sided test: p = 0.10, one-sided test “adults > children”: p = 0.05), indicating weaker urge-tic associations in children and adolescents compared to adults.

## Discussion

In this study, for the first time, urge-tic relationships were investigated using fine-grained, temporally specific methods in children and adolescents with GTS using the real-time urge monitor. At the group level, our analyses confirm an association between urges and tics, extending previous findings from adults^12,24^ to a developmental sample. As previously found in adults, inter-individual variability of urge-tic association patterns was high, with less than half of the participants showing a positive urge-tic association at the individual level. Across participants, higher tic intensities were associated with more pronounced urge-tic relationships, suggesting that anticipation of tics might be facilitated by more “prominent” tics. Compared to an adult sample^24^, the temporal association between urge and instantaneous tic intensity was found to be reduced in children and adolescents. This is consistent with the hypothesis that urges develop over time, presumably as an adaptation to tics, rather than being the direct cause of tics. However, this latter finding needs to be interpreted with caution as the group difference was only present in one of the three measures of urge-tic association.

In line with previous results in adult patients with GTS^12,24^, we found a positive association between urges and tics in children at the group level: stronger urges were associated with a greater likelihood to tic, urges were elevated around tic occurrence, and tic intensity correlated with urge. These results are consistent with the reported clinical phenomenology of premonitory urges in relation to tic execution^16,19,20,26^ and, at first sight, could be interpreted as support for the notion of urges causing tics. However, this interpretation is weakened by the high variability of individual urge-tic associations.

In fact, examining individual data, only between ten and twelve (40–48%; depending on the measure) children/adolescents showed a positive association between urge and tics while in eleven to 13 children/adolescents (44–52%), no significant correlation could be detected. In two (8%) patients, even a negative correlation was found. This heterogeneity is qualitatively and quantitatively comparable to previous findings in adult patients with GTS^24^, and as discussed there, is at odds with the notion of tics being directly caused by urges. Moreover, as argued in the introduction, assuming a causal relationship from urges to tics would also lead to the developmental prediction of more “pure” (pronounced and consistent) urge-tic associations, presumably less mitigated by voluntary tic suppression, in children and adolescents compared to adults. This prediction is incompatible with our finding of substantial heterogeneity in urge-tic associations in children and adolescents.

Comparison to data from a previously reported adult sample, using the same research methodology^24^, indicated weaker urge-tic associations in children and adolescents according to one of the three measures of urge-tic association (taking into account tic intensity). While the statistical strength of this result is limited, it is nevertheless informative with respect to the two opposing views concerning the nature of the relationship between urges and tics: as discussed in the introduction, tics in GTS are usually most severe during childhood^2,10,38^ and likely less influenced by voluntary suppression compared to adults. If tics were caused by urges, this would lead to the prediction of stronger, more “pure” urge-tic associations in childhood and adolescence compared to adults. In contrast, as we propose in the Introduction, if tics are the primary clinical phenomenon and urges develop as an adaptation to tics, for instance to better anticipate and possibly control one’s tics, urge-tic associations should become more pronounced during development. Our findings from the comparison to the adult sample do not support the first view but are, at least partially, consistent with the hypothesis of urges as an adaptation to tics.

Considering inter-individual differences in children and adolescents with GTS and the relation to clinical measures, we found a positive relation between tic frequency (tics/min) during a standardized video-based assessment (RUSH video protocol) and tic frequency (%) during the urge monitor recording. This result confirms good reliability and objectivity of the tic ratings. Consistent with prior findings, we also found a positive correlation between reported urge intensity in the urge monitor and total scores of the Premonitory Urge for Tics Scale (PUTS)^12,39^, documenting convergent validity with respect to the different urge measurements.

Similar to adults^24^, measures of the strength of urge-tic association did not show correlations with clinical scores, in particular not the Premonitory Urge to Tic Scale (PUTS)^40^. However, unlike adults^24^, each of the three measures of urge-tic association showed a significant positive correlation with mean tic intensity during the urge monitor. Higher tic intensities were associated with more pronounced positive urge-tic relationships, suggesting that anticipation of tics is facilitated by more “prominent” tics. This is consistent with the hypothesis that urges develop as an adaptation to tics: patients with stronger tics may be more likely to develop tic awareness as well as tic anticipation behavior, resulting in more pronounced urge-tic associations. Importantly (and as in the adult sample), measures of urge-tic associations did not correlate with mean or range (SD) of urge during the urge monitor, emphasizing that the detection of urge-tic associations was not confounded with participants’ use of the available urge scale.

Nine participants of the current sample indicated not experiencing premonitory urges in the clinical assessment. We decided not to exclude these participants from the study as the instructions of the urge monitor appeared to make sense to each of them and the urge monitor may provide additional, possibly more fine-grained information about participants’ premonitory sensations than a clinical questionnaire (PUTS). Of note, all of these nine participants indicated varying urge levels in the urge monitor and, importantly, most of them showed significant urge-tic associations for at least some of the measures of urge-tic association. Moreover, restricting the group-level analyses to these nine participants revealed significant urge-tic associations for two of the three analyses. This indicates that these patients, despite not reporting urges in the clinical interview, were able to indicate apparently meaningful urge levels in the urge monitor. A possible explanation of this mismatch could be a missing ability to verbally express or describe feelings of urges at young age although they are present^40–42^. Using a non-verbal outcome measure, as in the urge monitor, may facilitate the expression of subtle or vague premonitory sensations. This suggests that, also in epidemiological and in particular developmental studies, the assessment of premonitory urges should be based on multiple measurement instruments, ideally minimizing demands to verbally express one’s urges.

Considering neural mechanism, it appears that urges and tics emerge from distinct functional systems^43,44^. Cortico-basal ganglia-thalamo-cortical circuits and connected regions, particularly the basal ganglia, the primary motor cortex (M1) and medial frontal areas play a crucial role in tic generation and tic execution^43,45–48^. In contrast, primarily sensory regions including the primary and secondary somatosensory cortices have been implicated in the generation of urges^43–45,48–50^. These regions strongly project to the insula playing an important role in the integration of somatosensory, visceral and emotional information and interoception^45,50^. Both networks, i.e., tic generation circuits and loops generating urges are connected through pathways linking the insula, the supplementary motor area and the cingulate motor area, which in turn project to M1 and descending motor pathways^45,47,50^. These data suggest that urges and tic emerge from separate but interconnected neuronal networks. Interestingly, in a resting state functional connectivity MRI study it was found that in some of the connections between urge and tic networks physiological maturation including decreases in connectivity was delayed in patients with GTS patients, implying some degree of immaturity of these pathways in GTS^44^. These data could be interpreted such that supra-physiological connectivity between these areas leads to increased bodily awareness creating urges as a “byproduct” in GTS.

Limitations of the present study include the sample size and challenges of disambiguating tics from physiological movements. Based on established criteria, our current sample size is appropriate for detecting moderate to strong correlations and, in the comparison to the adult sample, allowed detecting large effect sizes. A larger sample size may have allowed detecting weaker or multi-factorial associations and group differences. As in the previously reported adult sample^24^, the heterogeneity in urge-tic associations provides evidence against a straightforward causal relation between urges and tics. However, performing the urge monitor depends on the capability of individuals to report their current urge level, making the task inherently variable. Thus, inter-individual variability in urge-tic associations may be partly explained by inter-individual differences in the ability to accurately sense and indicate their subjective urge intensity.

The ambiguity of tics within a spectrum of physiological movements^51^ results in a certain degree of uncertainty with respect to tic ratings, especially in children and adolescents, where distraction, “playing” or more generally the presence of “surplus movements” may render the distinction between tics and non-tic movements more difficult. This was addressed by preliminary viewing and agreement on non-tic-movements between the two raters prior to the detailed video rating (see Sect. 2.3 Video-based tic ratings). Moreover, urge-tic associations may be underestimated due to the fact that tics were only recorded and rated from the upper body while participants reported overall urge intensities which may also be related to tics occurring at the lower body^12^. Yet, inter-rater reliability scores and agreement with tic ratings during the RUSH protocol indicate good reliability and objectivity of the tic measures.

To minimize systematic differences between age groups, the raters for the present study were trained and supervised by the raters of the previously acquired adult sample^24^ until high inter-rater reliability was achieved between all four raters. Moreover, while the rating scheme of the present study is a subset of the rating scheme used in the adult study (which also included information on body region and tic onset), it does include all the relevant information used in the analysis of the adult sample (tic presence/absence and tic intensity). The validity of the measures from the urge monitor task might be different for adults as compared to children (e.g., children might be more prone to distraction). Before conducting the task, the importance of not “playing” but indicating the real current urge feeling was explained to the patients age-appropriately. All videos and data from the urge monitor task were checked for any indications of not executing the task properly. This led to the exclusion of one patient. In the data of the remaining patients there were no signs of not performing the instructed task.

Using an established self-report tool, the urge monitor, to capture premonitory urge intensities and determine urge-tic associations in GTS, our results confirm significant positive correlations between premonitory urges and tics also in children and adolescents with GTS. Substantial inter-individual variability in urge-tic associations as well as some evidence for weaker urge-tic associations in the present compared to a previous adult sample are incompatible with the notion of tics solely being a consequence of elevated urge but are consistent with the hypothesis of urges developing as an adaptation to tics, for instance to anticipate and control tic expression. These findings are of considerable importance for the understanding of GTS and are potentially also relevant for therapy. Existing behavioral treatments, such as the Comprehensive Behavioral Intervention for Tics (CBIT), train participants to perform competing (non-tic) actions when they feel the urge to execute a tic e.g., Ref.^52^. In children and adolescents without urges or only weak urge-tic associations, alternative strategies, e.g., attention training techniques, might be more appropriate^53^. Investigations of urge-tic relations may thus become useful for personalized treatment approached in this common neuropsychiatric disorder.

## Supplementary Information

Supplementary Information.

## Data Availability

Original data and analysis scripts are available from a public OSF repository (https://osf.io/zwnsc/).
