# Supplementary material for: Urge-tic associations in children and adolescents with Tourette syndrome
Source: Sci Rep. 2022 Sep 26;12:16008. doi: 10.1038/s41598-022-19685-5 (PMC9512906; doi:10.1038/s41598-022-19685-5)
Supplement: Supplementary file 1 — Supplementary Information. [file 41598_2022_19685_MOESM1_ESM.pdf]

Urge-tic associations in children and adolescents with Tourette syndrome

Supplementary Materials

Langelage et al (2022)

1 Clinical assessment and participant information

1.1 Clinical assessment

For the assessment of tic severity and frequency we used the Yale Global Tic Severity Scale (YGTSS) [1] (mean score ± SD: 27.7 ± 16.9, range 4-67, [of total scale 0-100]), the Diagnostic Confidence Index (DCI) [2] (43.6 ± 16.1, range 18-89, [of 0-100]), and the modified RUSH video protocol [3]. The RUSH video protocol offers a structured scoring method to analyze five domains (number of involved body regions, number of motor and vocal tics, intensity of motor and vocal tics) to calculate a global tic score and the tic frequency (tics/min). For the video protocol, two conditions are recorded: 1) relaxed, no tic suppression with the examiner in the room and 2) relaxed, no tic suppression in absence of the examiner. Ten minutes of video recording are divided in 4 parts, each 2.5 minutes. In the first and second part (with/without examiner in the room, respectively), the whole body of the participant is recorded from a distance, hands are placed on the knees in both conditions. During the third and fourth part, the upper body is filmed under the same conditions. Only the video sequences without the examiner in the room are used for the ratings. Similar to previous studies[4,5], all Rush videos were rated independently by two experts experienced in the assessment of GTS. When scores differed, relevant segments of the videos were reviewed and discussed to determine a RUSH consensus score. If tic counts differed by more than 15%, the count was repeated independently after this review process, which resulted in tic counts differing by less than 15% in all cases. Tic counts were then averaged between raters. We determined the total RUSH score (10.0 ± 3.61, range 4-17.5 [of 0-20]) and the tic frequency (tics per minute) (38.4 ± 25.7, range 4.4-104.0).

Furthermore, the GTS Quality of life Scale (GTS-QoL) [6] was applied investigating the impact GTS has on the quality of patients´ lives (14.4 ± 18.2, range 0-59.6 [of 0-100]). Participants from the

age of 11 reported the nature and severity of premonitory urges with the nine item version of the Premonitory Urge to Tic Scale (PUTS) [7] (15.0 ± 6.93, range 9-34 [of 9-36]). The PUTS was only administered to participants experiencing premonitory urges according to an initial screening question; the remaining participants were assigned the minimal possible PUTS score of 9. Symptoms of OCD were captured with the Children's Yale-Brown Obsessive-Compulsive Scale (CY-BOCS) in children and adolescents in the age range from 8 to 15 years [8] and with using the Yale-Brown Obsessive-Compulsive Scale (Y-BOCS) for adolescents from the age of 16 [9] (3.7 ± 5.9, range 0-18 [of 0-40]). ADHD was assessed using the Conners Child ADHD Rating Scale (third edition, short version C3K-self assessment) [10,11]. For correlation analysis, we used the sub-scores for hyperactivity/impetuosity (Conners HI, 53.6 ± 8.5, range 41-73) and inattentiveness (Conners IA, 51.6 ± 9.3, range 39-73, both standardized to mean 50 and SD 10).

A summary of clinical data is given in Table S1. An overview of the comparison of clinical characteristics between the current sample of children and adolescents and the adult sample [4] is provided in Table S2. Compared to the adult sample, children and adolescents scored lower on YGTSS, PUTS, GTS-QoL and DCI.

1. Leckman, J. F. *et al.* The Yale Global Tic Severity Scale: initial testing of a clinician-rated scale of tic severity. *J. Am. Acad. Child Adolesc. Psychiatry* **28**, 566–573 (1989).
2. Robertson, M. M. *et al.* The Tourette Syndrome Diagnostic Confidence Index: Development and clinical associations. *Neurology* **53**, 2108–2108 (1999).
3. Goetz, C. G., Pappert, E. J., Louis, E. D., Raman, R. & Leurgans, S. Advantages of a modified scoring method for the Rush Video-Based Tic Rating Scale. *Mov. Disord. Off. J. Mov. Disord. Soc.* **14**, 502–506 (1999).
4. Schubert, L. *et al.* Inter-individual differences in urge-tic associations in Tourette syndrome. *Cortex* (2021) doi:10.1016/j.cortex.2021.06.017.
5. Kleimaker, M. *et al.* Increased perception-action binding in Tourette syndrome. *Brain* **143**, 1934–1945 (2020).
6. Cavanna, A. E. *et al.* The Gilles de la Tourette Syndrome–Quality of Life Scale (GTS-QOL): Development and validation. *Neurology* **71**, 1410–1416 (2008).
7. Woods, D. W., Piacentini, J., Himle, M. B. & Chang, S. Premonitory Urge for Tics Scale (PUTS): initial psychometric results and examination of the premonitory urge phenomenon in youths with Tic disorders. *J. Dev. Behav. Pediatr. JDBP* **26**, 397–403 (2005).
8. Goodmann, W. K., Price, L. H., Rasmussen, S. A., Riddle, M. A. & Rapoport, J. L. CY-BOCS Beurteilungsskala für Zwangsstörungen bei Kindern. www.kjpd.uzh.ch/pdf/praxis (1993).

9. Goodman, W. K. *et al.* The Yale-Brown Obsessive Compulsive Scale: I. Development, Use, and Reliability. *Arch. Gen. Psychiatry* **46**, 1006–1011 (1989).
10. Conners, C. K., Pitkanen, J. & Rzepa, S. R. Conners 3rd Edition (Conners 3; Conners 2008). in *Encyclopedia of Clinical Neuropsychology* (eds. Kreutzer, J. S., DeLuca, J. & Caplan, B.) 675–678 (Springer, 2011). doi:10.1007/978-0-387-79948-3_1534.
11. Lidzba, K., Christiansen, H. & Drechsler, R. Conners-3. Conners Skalen zu Aufmerksamkeit und Verhalten – 3. Deutschsprachige Adaptation der Conners 3rd EditionTM (Conners 3TM) von C. Keith Conners. *Lidzba Karen Christ. Hanna Drechsl. Renate 2013 Conners-3 Conners Skalen Zu Aufmerksamkeit Verhalt. – 3 Deutschsprachige Adapt. Conners 3rd Ed. Conners 3TM Von C Keith Conners Bern Verl. Hans Huber* (2013).

1.2 Clinical data and participants information

Table S1: Participant information and clinical characteristics of Tourette patients (D = Dresden, L = Lübeck). Sex (female/male), Age (years), Modified RUSH video protocol (RUSH total score and tics/min), Yale Global Tic Severity Scale (YGTSS, total score), Premonitory Urge to Tic Scale (PUTS), Gilles de la Tourette Syndrome - Quality of Life Scale (GTS-QoL), Yale-Brown Obsessive-Compulsive Scale for children (CY-BOCS) and adults (Y-BOCS), Conners ADHD Rating Scale for children, subscales for inattentiveness (IA) and hyperactivity/impetuosity (HI), short form of the Wechsler Intelligence Scale for Children (HAWIK-IV) / short form of the Wechsler Adult Intelligence Scale - fourth Edition (WAIS-IV), GTS Diagnostic Confidence Index (GTS-DCI). Adult scales were used for participants of age 16 and above.

NOTE: The PUTS was only administered to patients above the age of 10 and indicating experiencing urges in a preliminary screening question. Patients responding "no" to the screening question (N=9) were assigned the minimal PUTS score (9, marked with a * below). Unfortunately, clinical scores are missing from a few additional participants for the PUTS (N=2), GTS-QoL (N=3), and Conners IA and HI (N=3).

ID	Sex	Age	RUSH (total)	RUSH (tics/min)	YGTSS	PUTS	GTS-QoL	CY-BOCS / YBOCS	Conners (IA)	Conners (HI)	HAWIK / WAIS (IQ)	GTS-DCI
D01	m	17	8.5	12.4	22	16	1.9	7	45	45	118	41
D02	f	18	8.0	27.8	29	15	2.8	0	42	52	88	51
D03	m	18	10.5	21.4	13			14			98	40
D04	m	16	9.0	15.6	9	18	1.9	2	55	42	108	36
D05	m	16	13.5	77.2	39	24	12.0	2	69	59	123	48
D06	m	16	7.0	30.8	4	9*		0			102	26
D07	m	11	4.0	9.4	28	9*	1.0	0	51	50	101	28
D08	m	16	4.0	4.4	4	9*	1.9	0	46	51	112	23
D09	m	13	11.0	58.6	24	19	9.6	0	44	52	93	18
D10	f	13	10.0	62.2	19		7.7	0	50	41	97	31
D11	f	15	17.5	63.6	67	34	57.7	17	62	68	121	89
D12	f	13	10.5	38.2	31	9*	0.0	0	50	52	110	34
D13	m	13	9.5	27.4	15	24	0.0	0	46	45	106	36
D14	f	14	7.5	31.8	5	9*	1.9	0	48	53	105	36
D15	f	8	4.0	8.8	37		7.7	0	41	62	91	43
L01	f	14	8.0	30.0	15	9*	45.2	13	67	44	110	42
L02	m	13	11.5	45.0	29	9*		0	46	57	108	41
L03	m	10	16.0	104.0	37		59.6	0	73	73	101	85
L04	m	12	16.0	64.8	59	9*	23.1	18	44	54	114	51
L05	f	11	14.0	83.0	44	17	24.0	7	52	64	113	48
L06	m	14	7.0	18.0	5	9*	4.8	8	59	62	117	52
L07	m	12	12.0	25.4	34	20	1.9	0	51	51	114	56
L08	m	9	12.0	44.0	40		26.9	0	55	58	97	44
L09	m	13	10.0	33.6	42	14	8.7	4	39	45	108	45
L10	m	17	9.0	22.0	42	18	17.6	0			89	46

Table S2: Comparison of clinical measures to data from the adult sample (Schubert et al, 2021) by two-sample Welch t-tests.

Clinical scores	Statistics	Children	Adults
**YGTSS**	**t(43.7)=-3.05, p=0.004**	**27.72 (16.92)**	**42.24 (15.30)**
RUSH (total)	t(41.4)=-1.73, p=0.091	10.00 (3.61)	11.52 (2.32)
RUSH_(tics/min)	t(43.8)=0.66, p=0.515	38.38 (25.67)	33.93 (20.20)
PUTS	t(35.8)=-3.19, p=0.003	15.00 (6.93)	21.19 (5.37)
**GTS-QoL**	**t(32.3)=-3.49, p=0.001**	**14.45 (18.20)**	**41.41 (30.59)**
**GTS-DCI**	**t(38.9)=-3.00, p=0.005**	**43.60 (16.13)**	**59.57 (19.45)**
CY-BOCS/YBOCS	t(38.5)=-1.64, p=0.108	3.68 (5.86)	6.90 (7.21)

2 Supplementary methods

2.1 Synchronization of urge and tic data

The times of these signals in the video recording were determined by visual inspection of the recorded sound waves (Audacity, version 2.2.3, https://audacityteam.org/), showing good agreement with the duration of the urge recording (deviation between -36ms and +136ms, with one exception where the deviation was 620ms). In nine videos (all from the University of Lübeck), the audio signal was not recorded due to technical problems. In these cases, video and urge data were synchronized by means of file system time stamps of the recordings and log file timing data. Applying this procedure to 20 independent recordings from the same setup with known (audio-based) synchronization, resulted in deviations of -0.10s +/- 0.59s (range: -0.79s - 1.54s). Furthermore, screen luminance abruptly changes at the end of the urge monitor task, the reflection of which could often be detected in the video recording. We only included videos in the analysis where the luminance change could be clearly detected and did not deviate by more than 0.5s from the synchronization estimated from file time stamps. As reported urge usually varies at considerably longer time scales (5-10s), we consider this inaccuracy acceptable.

2.2 Tic rating scales

Table S3: Tic intensity rating scales for motor and vocal tics.

Intensity	Motor	Vocal
**0**	None	None
**1**	Very mild; could be a normal movement (e.g., short sniff)	Very mild; could be a spontaneous physiological sound (e.g., throat clearing; single)
**2**	Mild, clearly tic; undoubtedly noticeable to tic expert but may not be noticed by others (e.g., nasal flare; opening eyes)	Mild, probably tic (e.g., throat clearing repetitive)
**3**	Mild, clearly tic; noticeable to any observer attending to it, no exaggerated muscle activity (e.g., mouth pouting, kiss)	Mild clearly tic; noticeable to any observer attending to it (e.g., grunt, shriek, cheep; single)
**4**	Moderate; easily noticeable, exaggerated muscle activity (e.g., screwing up the eyes and raising the nose)	Moderate; easily noticeable, sounds, no words (e.g., bark)
**5**	Moderate to severe; exaggerated muscle activity leading to unmissable distortions (e.g., tension throughout the whole body)	Moderate; easily noticeable, single non-coprolalic words (e.g., "hello!", "haha!")
**6**	Severe; unusual movement clearly raising attention (e.g., cramped fist knocking on the table)	Moderate to severe; single coprolalic words (e.g., "fuck!")
**7**	Severe; unusual movement that is off-putting or frightening (e.g., mouth wide open and cramped middle finger pointing)	Severe; multiple coprolalic words, noisy sound (e.g., yelling)
**8**	Very severe, i.e., apparently painful or potentially dangerous, e.g., due to self-injury (hitting in the face)	

3 Individual urge and tic data

Table S4: Individual urge and tic data of Tourette patients during the urge monitor, including tic frequency (percentage of one-second intervals with rated tics), mean tic intensity, mean (SD) and range of urge intensity.

ID	Tic frequency (%)	Tic intensity	Urge mean (SD)	Urge range
D01	45.7	1.8	1.6 (6.0)	0.0 - 50.0
D02	61.3	2.3	1.7 (7.5)	0.0 - 50.0
D03	71.7	2.3	18.8 (20.8)	0.0 - 83.8
D04	19.3	1.5	1.5 (6.5)	0.0 - 50.0
D05	93.0	3.4	58.2 (45.8)	0.0 - 100.0
D06	43.0	1.3	1.4 (3.2)	0.0 - 33.6
D07	41.7	2.0	0.1 (0.8)	0.0 - 12.7
D08	23.3	1.7	0.0 (0.3)	0.0 - 6.7
D09	66.3	2.3	21.4 (23.1)	0.0 - 100.0
D10	62.0	2.3	1.9 (6.2)	0.0 - 44.0
D11	85.7	2.9	39.7 (36.6)	0.0 - 100.0
D12	76.0	2.3	35.6 (38.0)	0.0 - 100.0
D13	59.3	2.1	6.3 (15.4)	0.0 - 76.1
D14	45.3	2.2	2.3 (5.1)	0.0 - 50.0
D15	10.7	2.2	0.5 (3.2)	0.0 - 48.5
L01	83.3	2.3	7.7 (0.9)	6.0 - 12.0
L02	53.7	2.4	0.1 (0.6)	0.0 - 8.2
L03	79.3	2.2	39.8 (33.5)	0.0 - 100.0
L04	63.3	2.8	0.2 (1.4)	0.0 - 24.4
L05	92.7	3.0	23.9 (24.8)	0.0 - 100.0
L06	28.7	1.8	0.1 (0.6)	0.0 - 5.5
L07	98.3	2.5	2.0 (6.7)	0.0 - 33.1
L08	84.3	2.7	28.7 (33.2)	0.0 - 100.0
L09	84.7	2.3	21.2 (30.1)	0.0 - 100.0
L10	63.3	1.9	9.6 (21.9)	0.0 - 89.3

4 Individual urge-tic associations

Table S5: Statistical results from the urge-tic analyses at the level of individual participants: logistic regression (predicting tic occurrence from urge), linear regression (predicting urge from tic) and correlation (between urge and instantaneous tic intensity). Results with p<0.05 (uncorrected) are printed in bold font.

	Logistic regression			Linear regression			Correlation	
**ID**	**β**	**$\chi^2(1)$**	**p**	**β**	**t (278)**	**p**	**r**	**p**
D01	**0.49**	**9.74**	**0.002**	**0.34**	**2.91**	**0.004**	**0.36**	**< 0.001**
D02	**1.39**	**20.45**	**< 0.001**	**0.42**	**3.50**	**0.001**	**0.25**	**< 0.001**
D03	0.07	0.26	0.611	0.07	0.50	0.615	**0.16**	**0.008**
D04	-0.28	1.69	0.193	-0.17	-1.13	0.260	0.07	0.228
D05	**2.57**	**39.38**	**< 0.001**	**1.29**	**6.06**	**< 0.001**	**0.68**	**< 0.001**
D06*	-0.13	1.04	0.309	-0.12	-1.00	0.320	**-0.26**	**< 0.001**
D07*	0.16	1.68	0.195	0.16	1.29	0.197	0.05	0.435
D08*	**0.42**	**8.23**	**0.004**	**0.43**	**3.10**	**0.002**	0.11	0.062
D09	0.18	1.95	0.162	0.17	1.38	0.169	0.09	0.130
D10	**0.36**	**5.36**	**0.021**	**0.26**	**2.14**	**0.033**	**0.13**	**0.026**
D11	0.17	0.89	0.346	0.16	0.93	0.354	**0.33**	**< 0.001**
D12*	**0.61**	**14.80**	**< 0.001**	**0.52**	**3.76**	**< 0.001**	**0.27**	**< 0.001**
D13	0.12	0.85	0.355	0.11	0.91	0.366	-0.03	0.593
D14*	**0.40**	**7.70**	**0.006**	**0.31**	**2.63**	**0.009**	**0.23**	**< 0.001**
D15	0.30	4.56	0.033	**0.61**	**3.02**	**0.003**	-0.01	0.900
L01*	0.20	1.56	0.211	0.19	1.23	0.220	0.10	0.090
L02*	0.17	1.83	0.176	0.16	1.32	0.189	**0.13**	**0.030**
L03	**-0.68**	**21.53**	**< 0.001**	**-0.67**	**-4.84**	**< 0.001**	**-0.45**	**< 0.001**
L04*	**2.01**	**12.29**	**< 0.001**	0.24	1.91	0.057	**0.25**	**< 0.001**
L05	**2.34**	**29.17**	**< 0.001**	**0.95**	**4.43**	**< 0.001**	**0.63**	**< 0.001**
L06*	0.24	3.39	0.066	0.25	1.94	0.054	-0.04	0.495
L07				0.33	0.73	0.464	0.05	0.448
L08	0.11	0.39	0.531	0.10	0.62	0.537	0.06	0.325
L09	**-0.48**	**9.99**	**0.002**	**-0.55**	**-3.40**	**0.001**	0.00	0.976
L10	**0.99**	**30.58**	**< 0.001**	**0.60**	**5.09**	**< 0.001**	**0.22**	**< 0.001**

NOTE: Logistic regression model did not converge for L07. Participants not reporting/experiencing urges in the clinical assessment (PUTS 9) are marked with *.

5 Interindividual differences / correlation analyses

Complete results from the correlation analyses are reported below in tables. Scatter plots are shown for the correlations significant or marginally significant associations between urge, tic and urge-tic measures from the urge monitor and clinical/demographic scores.

5.1 Tics

Table S6: Correlation between tic measures from the urge monitor and clinical/demographic measures, with p-values corrected for the number of comparisons per row (two).

Measure	Tic frequency (%)	Tic intensity
Age	r=-0.09, p_{adj}=0.678	r=-0.27, p_{adj} =0.391
Disease duration	r=-0.04, p_{adj} =0.851	r=-0.17, p_{adj} =0.851
**RUSH (total)**	**r=0.72, p_{adj} <0.001**	**r=0.68, p_{adj} =<0.001**
**RUSH_(tics/min.)**	**r=0.62, p_{adj} =0.001**	**r=0.64, p_{adj} =0.001**
**YGTSS**	**r=0.54, p_{adj} =0.005**	**r=0.70, p_{adj} =<0.001**
PUTS	r=0.45, p_{adj} =0.097	r=0.43, p_{adj} =0.097
GTS-QoL	r=0.47, p_{adj} =0.056	r=0.39, p_{adj} =0.075
CY-BOCS/YBOCS	r=0.23, p_{adj} =0.268	r=0.34, p_{adj} =0.200
Conners (IA)	r=0.36, p_{adj} =0.196	r=0.25, p_{adj} =0.262
Conners (HI)	r=0.18, p_{adj} 0.416	r=0.43, p_{adj} =0.095

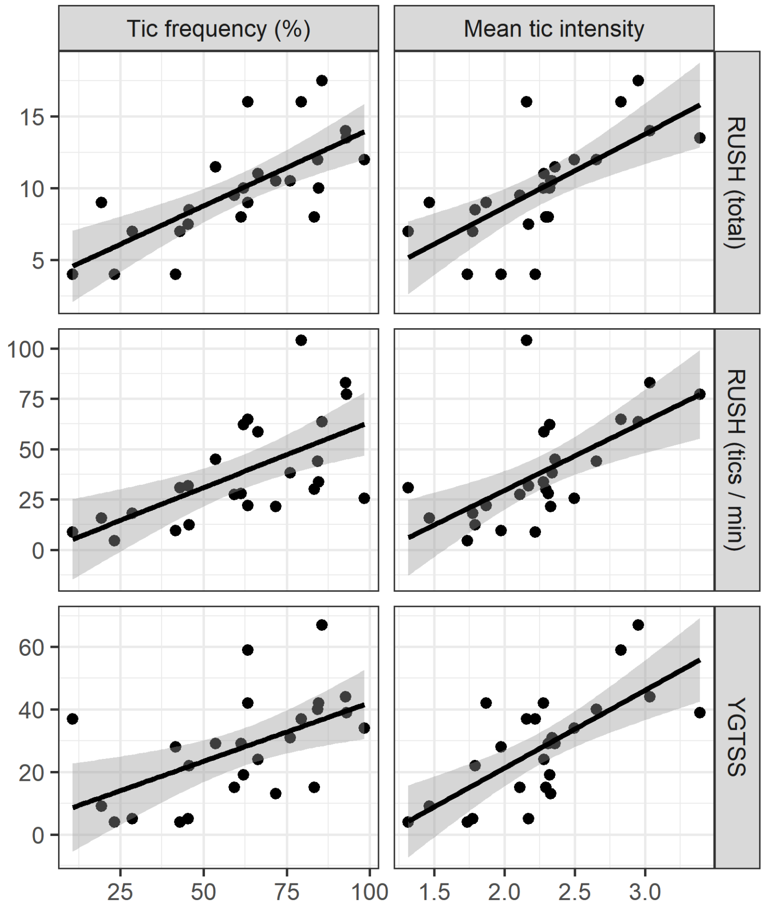

Tic frequency (%)
Mean tic intensity
RUSH (total)
RUSH (tics / min)
YGTSS
15
10
5
100
75
50
25
0
60
40
20
0
25
50
75
100
1.5
2.0
2.5
3.0

5.2 Urge

Table S7: Correlation between urge measures from the urge monitor and clinical/demographic measures, with p-values corrected for the number of comparisons per row (two).

Measure	Urge (mean)	Urge (SD)
Age	r=-0.08, p_{adj} =1.000	r=-0.07, p_{adj} =1.000
Disease duration	r=-0.07, p_{adj} =1.000	r=-0.06, p_{adj} =1.000
**RUSH (total)**	**r=0.63, p_{adj} =0.001**	**r=0.61, p_{adj} =0.001**
**RUSH (tics/min)**	**r=0.66, p_{adj} =0.001**	**r=0.58, p_{adj} =0.003**
**YGTSS**	**r=0.48, p_{adj} =0.016**	**r=0.52, p_{adj} =0.016**
**PUTS**	**r=0.56, p_{adj} =0.011**	**r=0.61, p_{adj} =0.009**
GTS-QoL	r=0.50, p_{adj} =0.036	r=0.39, p_{adj} =0.074
CY-BOCS/YBOCS	r=0.10, p_{adj} =1.000	r=0.01, p_{adj} =1.000
Conners (IA)	r=0.57, p_{adj} =0.010	r=0.39, p_{adj} =0.071
Conners (HI)	r=0.50, p_{adj} =0.035	r=0.41, p_{adj} =0.059
**Tic frequency (%)**	**r=0.65, p_{adj} =0.001**	**r=0.66, p_{adj} =0.001**
**Tic intensity**	**r=0.62, p_{adj} =0.002**	**r=0.56, p_{adj} =0.004**

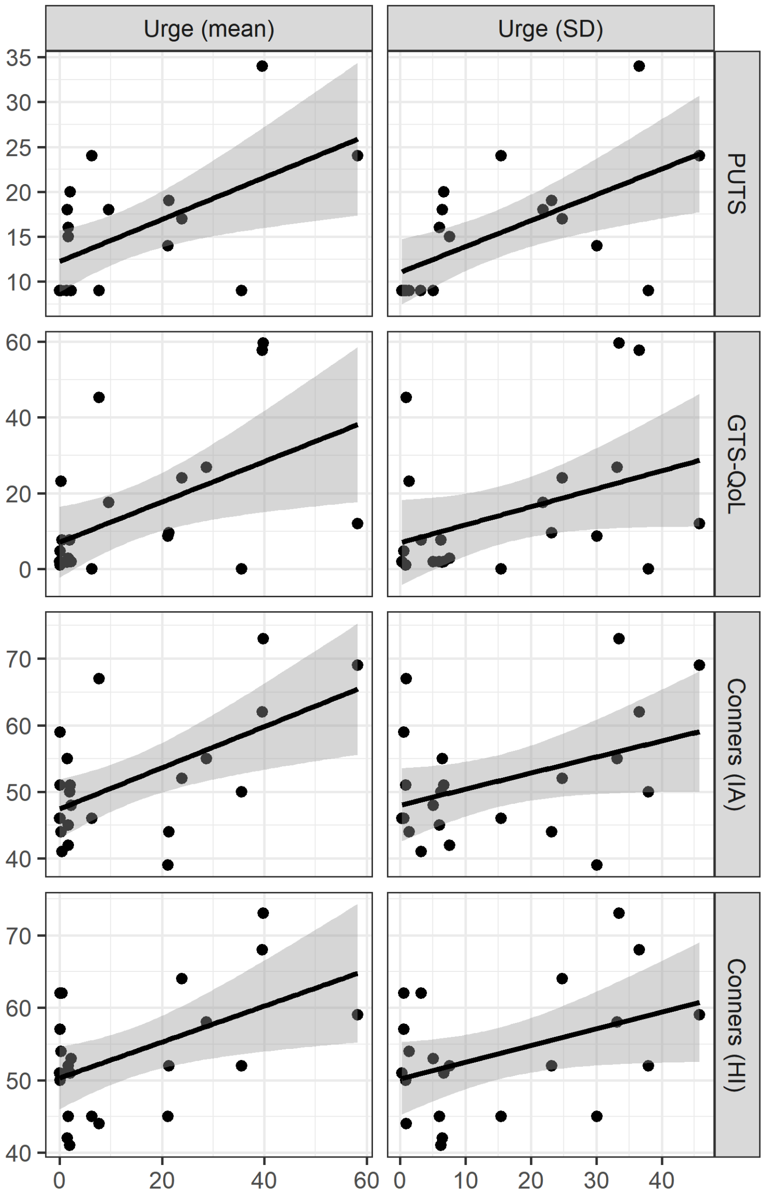

Urge (mean)
Urge (SD)
PUTS
GTS-QoL
Conners (IA)
Conners (HI)
35
30
25
20
15
10
60
40
20
0
70
60
50
40
70
60
50
40
0
20
40
60
0
10
20
30
40

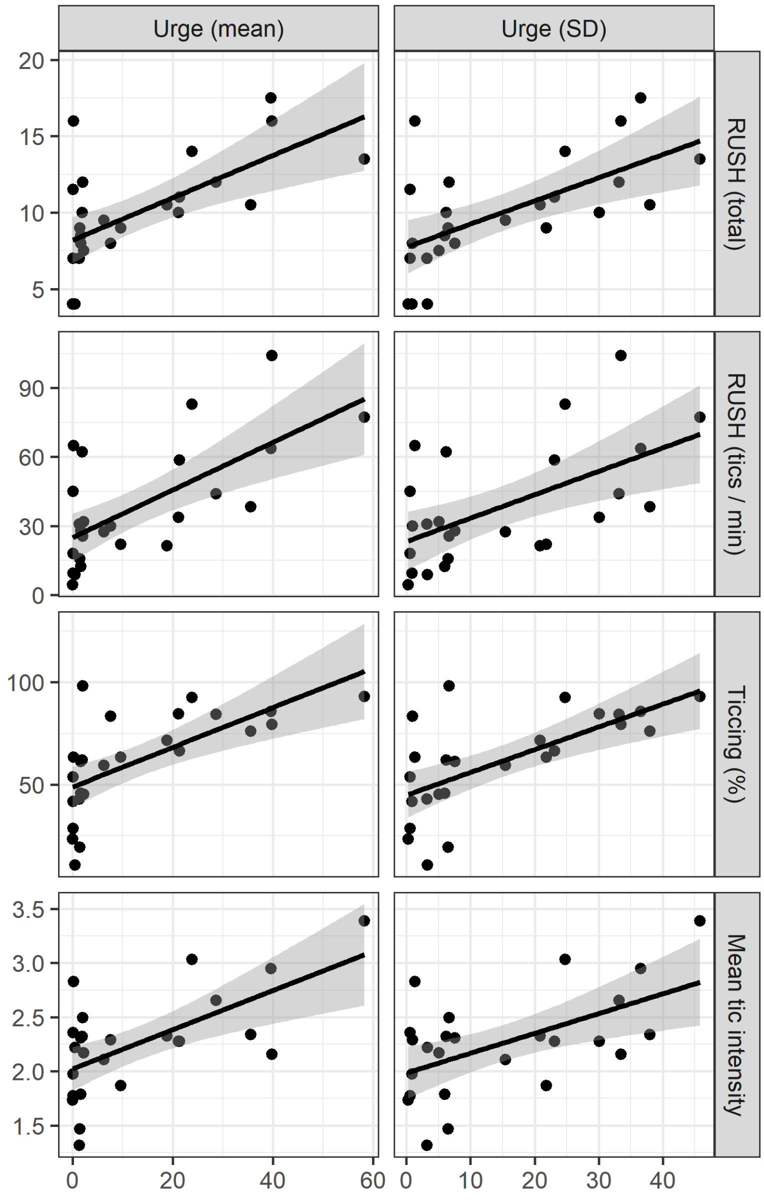

Urge (mean)
Urge (SD)
RUSH (total)
RUSH (tics / min)
Ticcing (%)
Mean tic intensity

5.3 Urge-tic association

Table S8: Correlation between measures of urge-tic association from the urge monitor and clinical/demographic measures, as well as urge and tic measures from the urge monitor, with p-values corrected for the number of comparisons per row (three).

Measure	Logistic regression	Linear regression	Correlation
Age	r=0.13, p_{adj} =1.000	r=0.12, p_{adj} =1.000	r=0.29, p_{adj} =0.483
Disease duration	r=0.03, p_{adj} =1.000	r=0.02, p_{adj} =1.000	r=-0.11, p_{adj} =1.000
RUSH_total_score	r=0.26, p_{adj} =0.640	r=-0.06, p_{adj} =0.779	r=0.23, p_{adj} =0.640
RUSH_tic/min.	r=0.31, p_{adj} =0.438	r=0.01, p_{adj} =0.987	r=0.14, p_{adj} =0.987
YGTSS	r=0.37, p_{adj} =0.220	r=0.14, p_{adj} =0.499	r=0.33, p_{adj} =0.225
PUTS	r=0.13, p_{adj} =0.875	r=0.18, p_{adj} =0.875	r=0.35, p_{adj} =0.381
GTS-QoL	r=-0.10, p_{adj} =1.000	r=-0.27, p_{adj} =0.652	r=-0.15, p_{adj} =1.000
CY-BOCS/YBOCS	r=0.20, p_{adj} =0.715	r=-0.02, p_{adj} =0.928	r=0.28, p_{adj} =0.520
Conners (IA)	r=-0.00, p_{adj} =1.000	r=-0.01, p_{adj} =1.000	r=-0.05, p_{adj} =1.000
Conners (HI)	r=0.15, p_{adj} =1.000	r=0.10, p_{adj} =1.000	r=-0.04, p_{adj} =1.000
Urge mean	r=0.20, p_{adj} =0.678	r=0.15, p_{adj} =0.678	r=0.29, p_{adj} =0.491
Urge SD	r=0.15, p_{adj} =0.968	r=0.09, p_{adj} =0.968	r=0.27, p_{adj} =0.578
Tic frequency (%)	r=0.30, p_{adj} =0.441	r=0.08, p_{adj} =0.720	r=0.30, p_{adj} =0.441
**Tic intensity**	**r=0.62, p_{adj} =0.003**	**r=0.46, p_{adj} =0.020**	**r=0.61, p_{adj} =0.003**

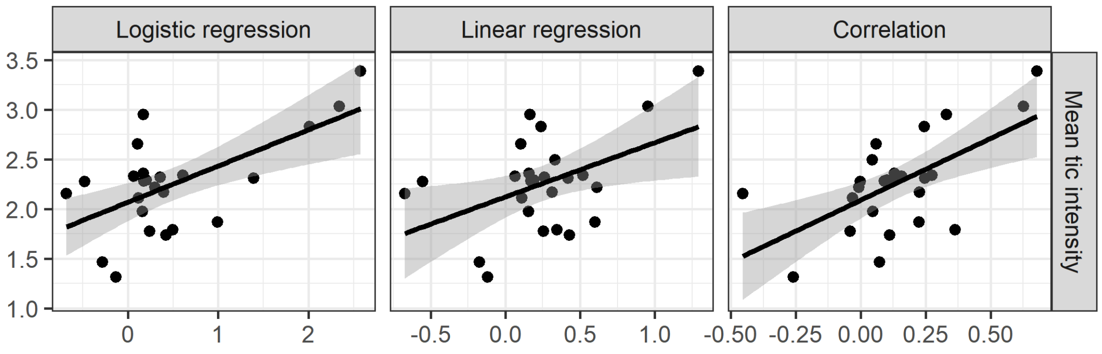

6 Comparison of age groups

Table S9: Comparison of clinical measures to data from the adult sample (Schubert et al, 2021) by two-sample Welch t-tests.

Urge and tic measures	Statistics	Children	Adults
**Tic frequency (%)**	**t(43.7)=2.18, p=0.035**	**61.44 (24.42)**	**47.57 (18.68)**
**Tic intensity**	**t(39.1)=-2.95, p=0.005**	**2.26 (0.48)**	**2.72 (0.57)**
Urge mean	t(43.7)=0.38, p=0.706	12.97 (16.42)	11.34 (12.71)
Urge SD	t(41.2)=1.07, p=0.289	14.89 (14.49)	11.10 (9.19)
Logistic regression	t(41.5)=0.28, p=0.779	0.49 (0.82)	0.43 (0.59)
Linear regression	t(42.8)=-0.84, p=0.404	0.25 (0.40)	0.35 (0.40)
**Correlation**	**t(38.2)=-1.69, p=0.100**	**0.13 (0.23)**	**0.27 (0.29)**

7 Individual urge-tic time series

For each data set included in the analysis, we present time series with reported urge (blue line), manually rated tics (vertical lines) and instantaneous tic intensity (central moving average of tic intensity, window size 11s; black line). Urge and tic intensity are standardized to mean 0 and standard deviation 1 (z-score).

ID D01

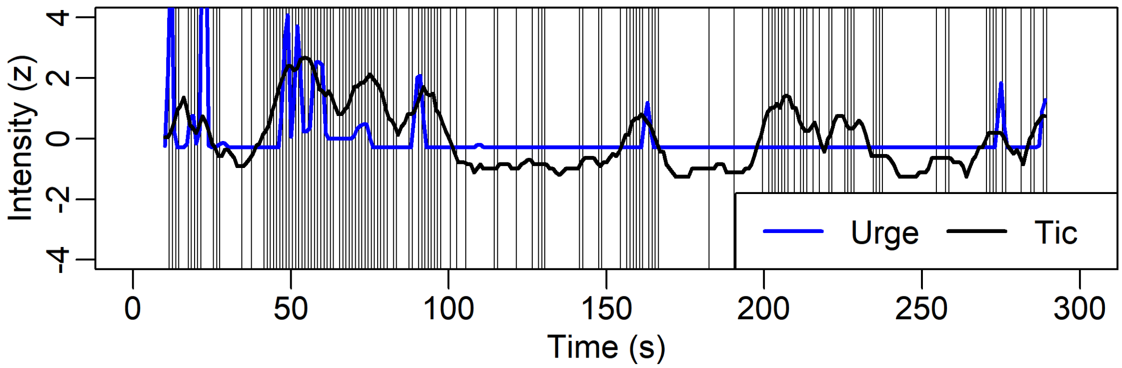

ID D02

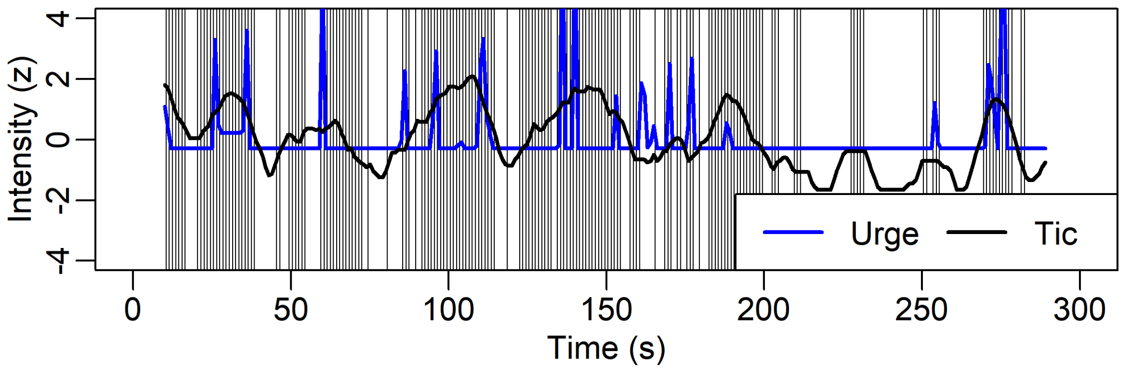

ID D03

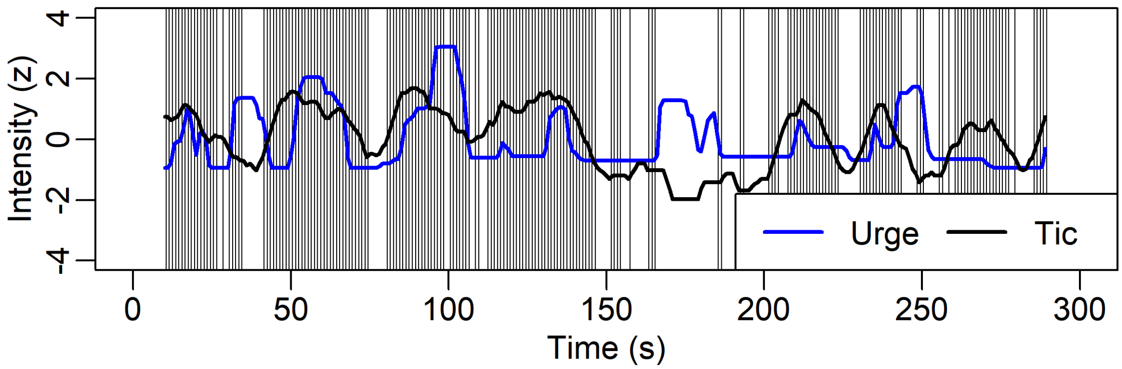

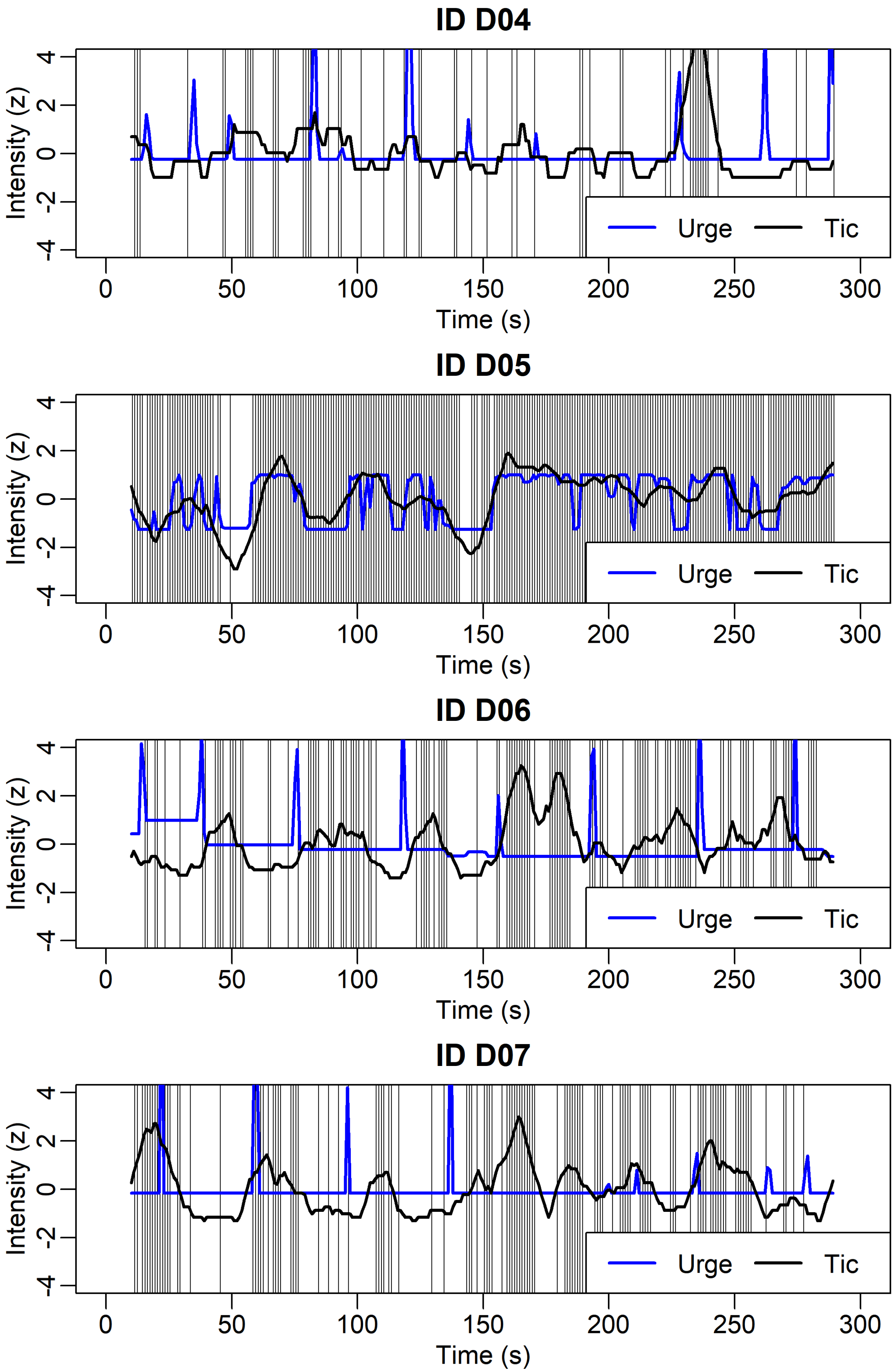
ID D04
ID D05
ID D06
ID D07
Intensity (z)
Time (s)
Urge
Tic
4
2
0
-2
-4
0
50
100
150
200
250
300

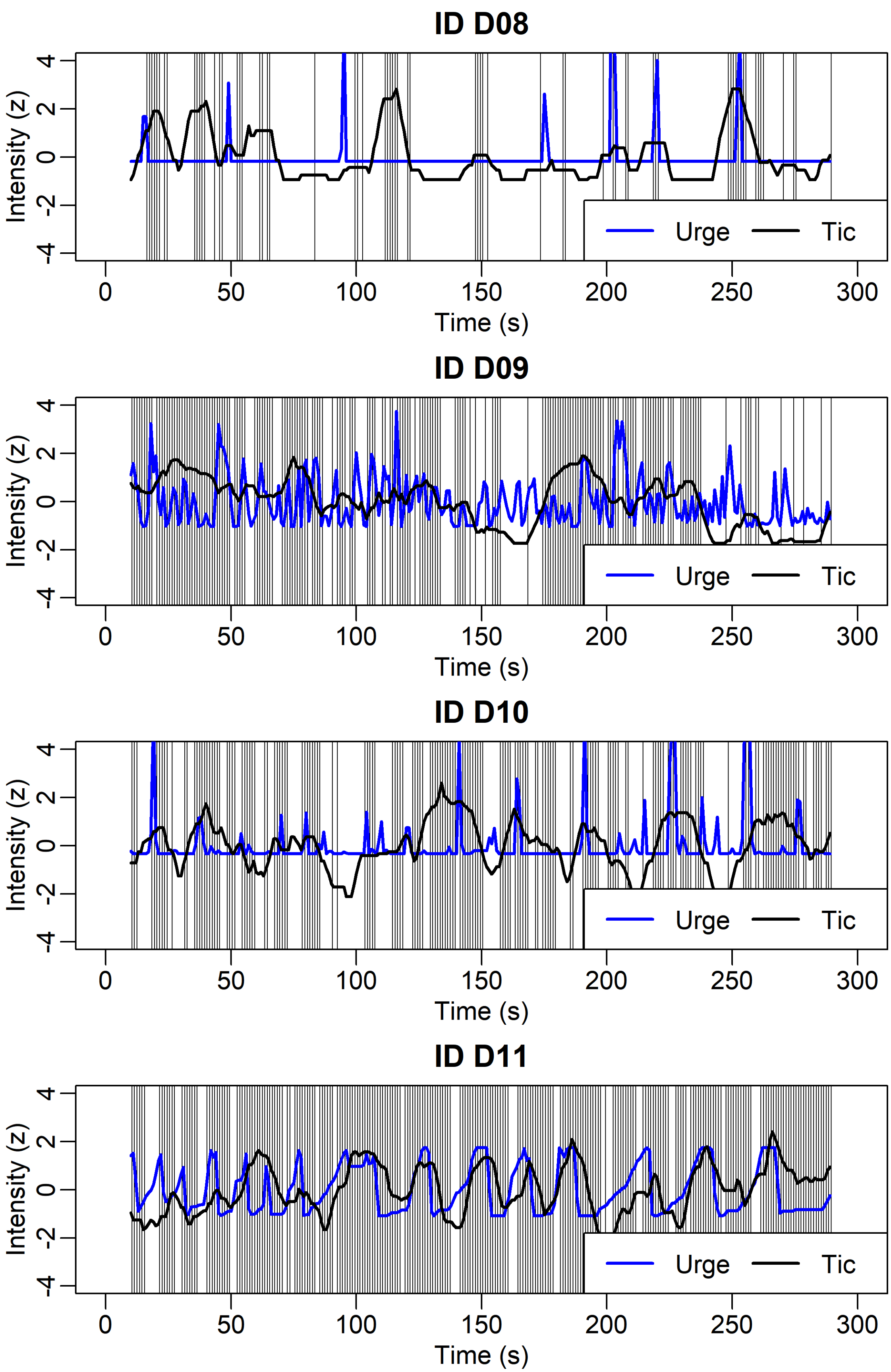

ID D08
Intensity (z)
Time (s)
Urge
Tic
ID D09
Intensity (z)
Time (s)
Urge
Tic
ID D10
Intensity (z)
Time (s)
Urge
Tic
ID D11
Intensity (z)
Time (s)
Urge
Tic

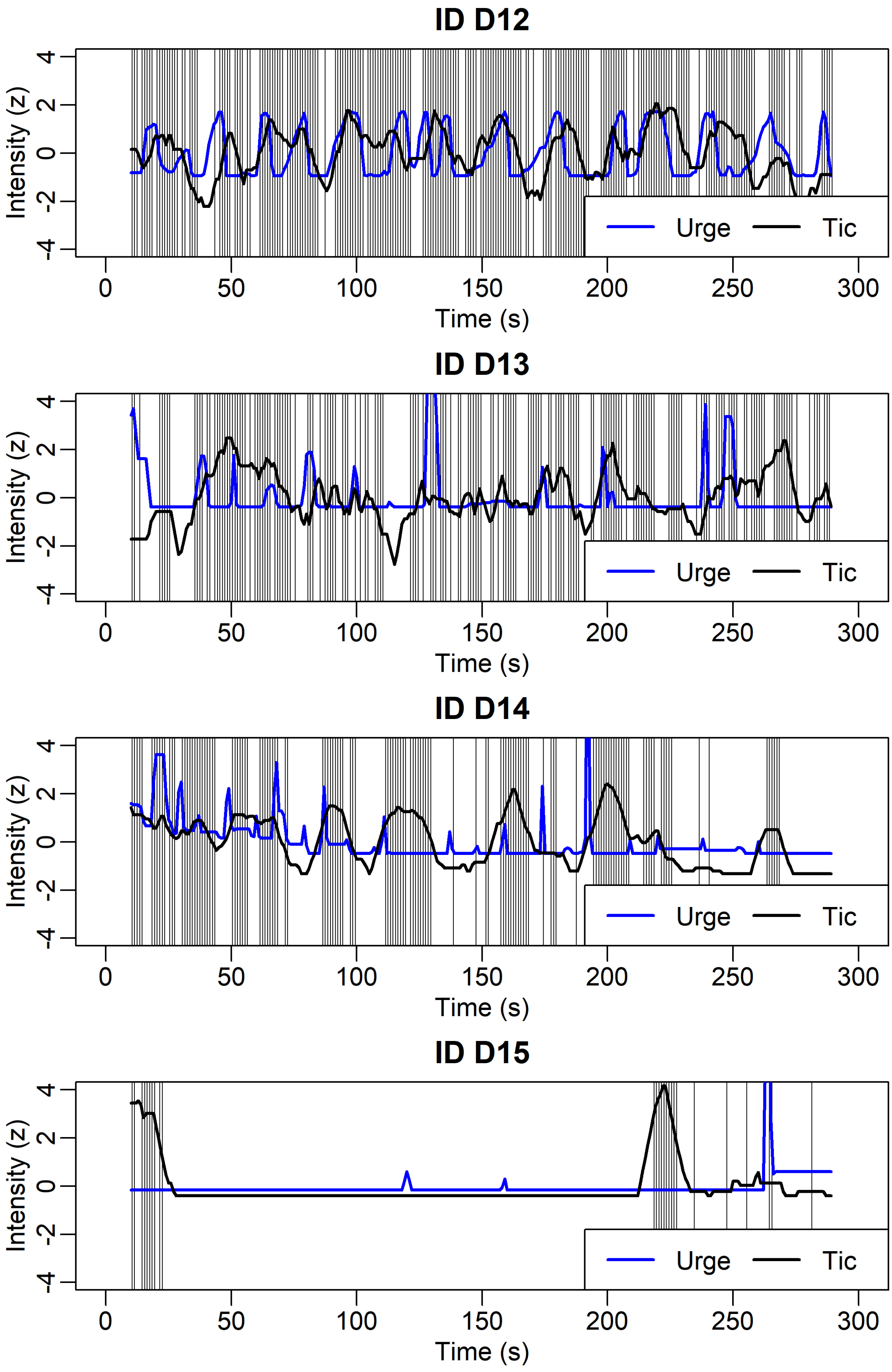
ID D12
ID D13
ID D14
ID D15
Intensity (z)
Time (s)
Urge
Tic
-4
-2
0
2
4
0
50
100
150
200
250
300

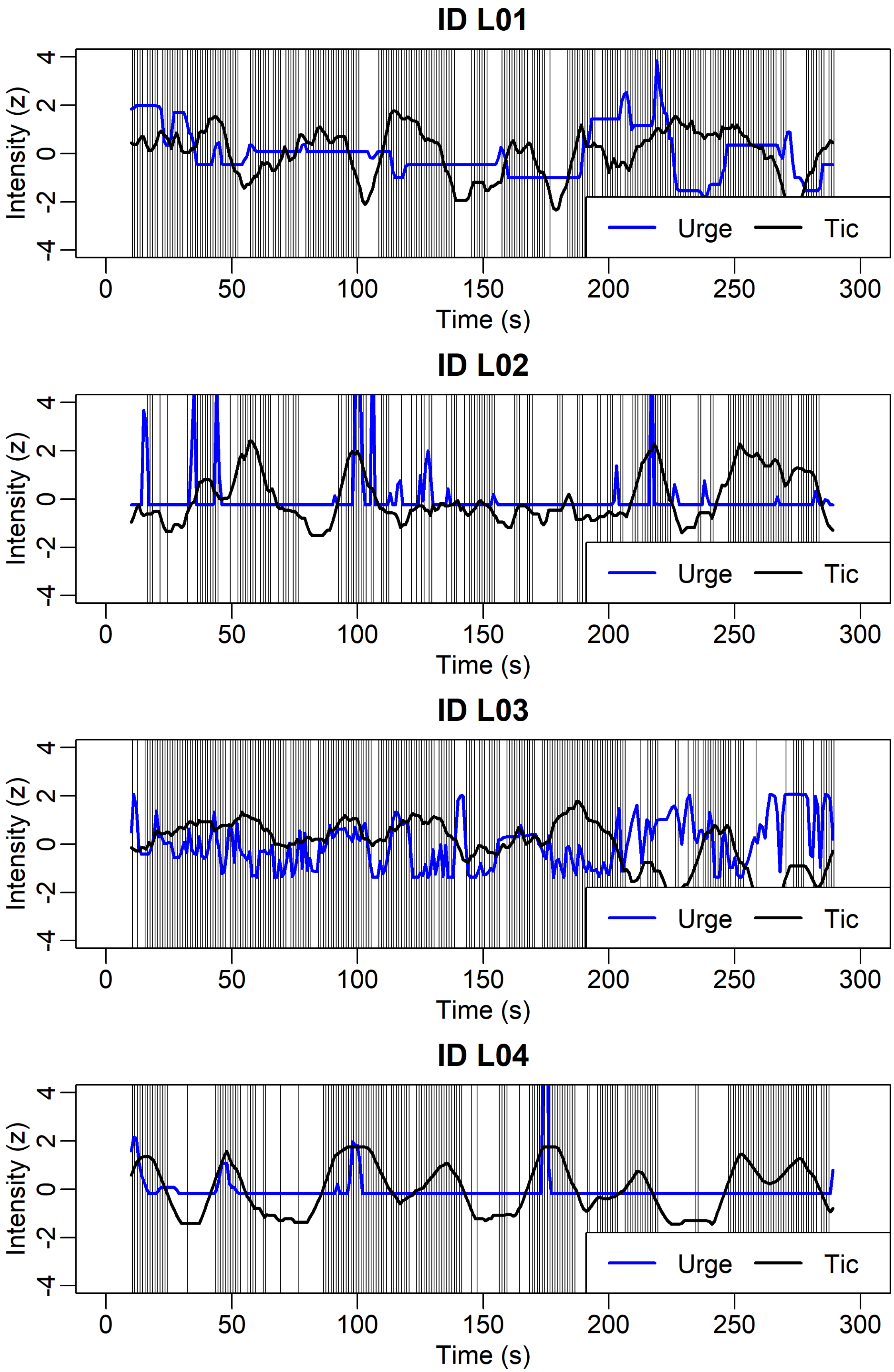
ID L01
Intensity (z)
Time (s)
Urge
Tic
ID L02
Intensity (z)
Time (s)
Urge
Tic
ID L03
Intensity (z)
Time (s)
Urge
Tic
ID L04
Intensity (z)
Time (s)
Urge
Tic

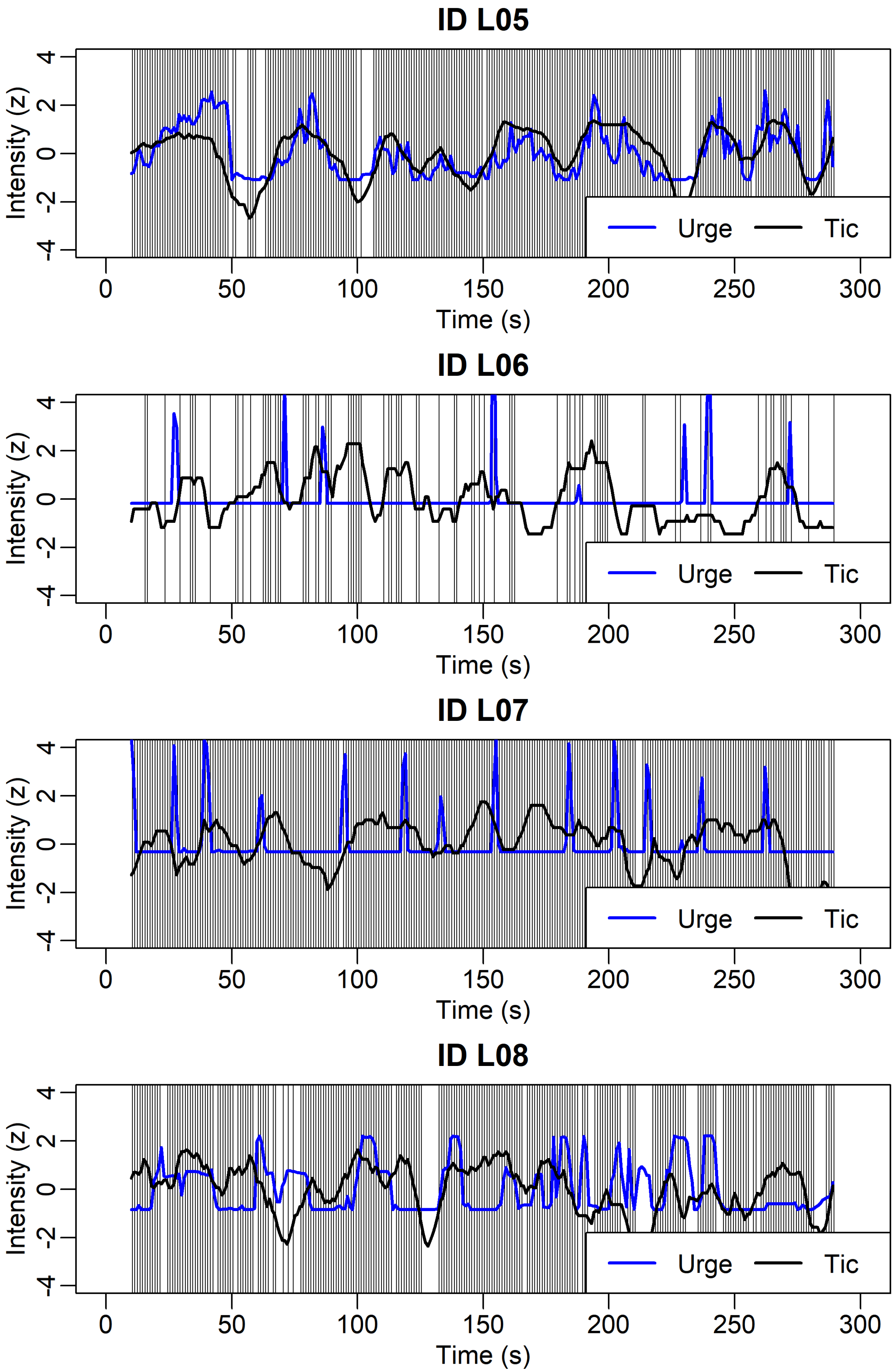
ID L05
ID L06
ID L07
ID L08
Intensity (z)
Time (s)
Urge
Tic
-4
-2
0
2
4
0
50
100
150
200
250
300

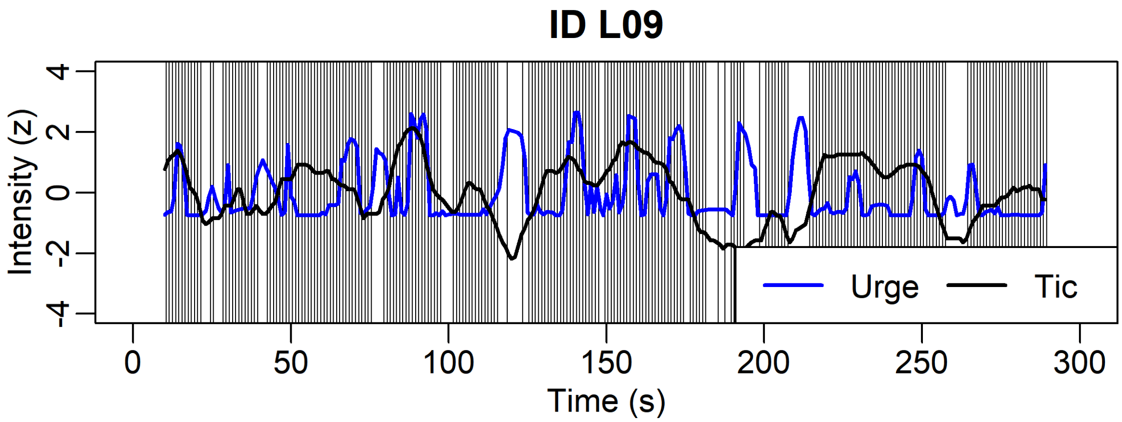

ID L09
Intensity (z)
4
2
0
-2
-4
0
50
100
150
200
250
300
Time (s)
Urge
Tic

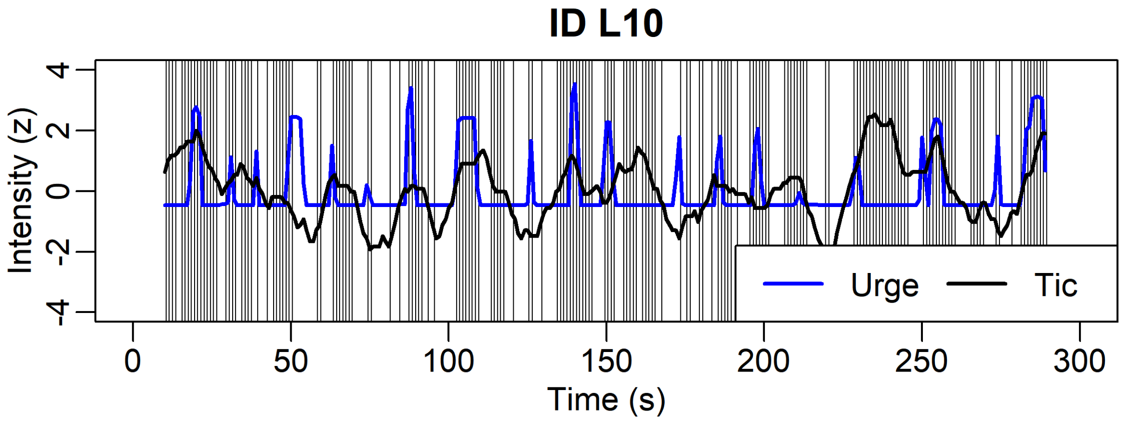

ID L10
Intensity (z)
4
2
0
-2
-4
0
50
100
150
200
250
300
Time (s)
Urge
Tic
